# A Quantitative Comparison of Human HT-1080 Fibrosarcoma Cells and Primary Human Dermal Fibroblasts Identifies a 3D Migration Mechanism with Properties Unique to the Transformed Phenotype

**DOI:** 10.1371/journal.pone.0081689

**Published:** 2013-12-03

**Authors:** Michael P. Schwartz, Robert E. Rogers, Samir P. Singh, Justin Y. Lee, Samuel G. Loveland, Justin T. Koepsel, Eric S. Witze, Sara I. Montanez-Sauri, Kyung E. Sung, Emi Y. Tokuda, Yasha Sharma, Lydia M. Everhart, Eric H. Nguyen, Muhammad H. Zaman, David J. Beebe, Natalie G. Ahn, William L. Murphy, Kristi S. Anseth

**Affiliations:** 1 Department of Biomedical Engineering, University of Wisconsin-Madison, Madison, Wisconsin, United States of America; 2 Department of Orthopedics and Rehabilitation, University of Wisconsin-Madison, Madison, Wisconsin, United States of America; 3 Materials Science Program, University of Wisconsin-Madison, Madison, Wisconsin, United States of America; 4 Paul P. Carbone Comprehensive Cancer Center, University of Wisconsin-Madison, Madison, Wisconsin, United States of America; 5 Laboratory for Optical and Computational Instrumentation, University of Wisconsin-Madison, Madison, Wisconsin, United States of America; 6 Department of Chemical and Biological Engineering, University of Colorado at Boulder, Boulder, Colorado, United States of America; 7 Department of Chemistry and Biochemistry, University of Colorado at Boulder, Boulder, Colorado, United States of America; 8 Howard Hughes Medical Institute, University of Colorado at Boulder, Boulder, Colorado, United States of America; 9 Department of Cancer Biology, Perelman School of Medicine, University of Pennsylvania, Philadelphia, Pennsylvania, , United States of America; 10 Department of Biomedical Engineering, Boston University, Boston, Massachusetts, United States of America; 11 College of Medicine, Texas A&M Health Science Center, Bryan, Texas, United States of America; 12 Department of Chemical and Materials Engineering, University of Dayton, Dayton, Ohio, United States of America; Fox Chase Cancer Center, United States of America

## Abstract

Here, we describe an engineering approach to quantitatively compare migration, morphologies, and adhesion for tumorigenic human fibrosarcoma cells (HT-1080s) and primary human dermal fibroblasts (hDFs) with the aim of identifying distinguishing properties of the transformed phenotype. Relative adhesiveness was quantified using self-assembled monolayer (SAM) arrays and proteolytic 3-dimensional (3D) migration was investigated using matrix metalloproteinase (MMP)-degradable poly(ethylene glycol) (PEG) hydrogels (“synthetic extracellular matrix” or “synthetic ECM”). In synthetic ECM, hDFs were characterized by vinculin-containing features on the tips of protrusions, multipolar morphologies, and organized actomyosin filaments. In contrast, HT-1080s were characterized by diffuse vinculin expression, pronounced β1-integrin on the tips of protrusions, a cortically-organized F-actin cytoskeleton, and quantitatively more rounded morphologies, decreased adhesiveness, and increased directional motility compared to hDFs. Further, HT-1080s were characterized by contractility-dependent motility, pronounced blebbing, and cortical contraction waves or constriction rings, while quantified 3D motility was similar in matrices with a wide range of biochemical and biophysical properties (including collagen) despite substantial morphological changes. While HT-1080s were distinct from hDFs for each of the 2D and 3D properties investigated, several features were similar to WM239a melanoma cells, including rounded, proteolytic migration modes, cortical F-actin organization, and prominent uropod-like structures enriched with β1-integrin, F-actin, and melanoma cell adhesion molecule (MCAM/CD146/MUC18). Importantly, many of the features observed for HT-1080s were analogous to cellular changes induced by transformation, including cell rounding, a disorganized F-actin cytoskeleton, altered organization of focal adhesion proteins, and a weakly adherent phenotype. Based on our results, we propose that HT-1080s migrate in synthetic ECM with functional properties that are a direct consequence of their transformed phenotype.

## Introduction

To successfully metastasize, tumor cells must leave the primary tumor and then navigate numerous tissue barriers before establishing secondary tumors at distant sites [1-3], motivating efforts to elucidate mechanisms of 3-dimensional (3D) migration and invasion [4-14]. Tumor cells have been characterized by the capacity to transition between distinct migration modes in 3D culture [4,5], an inherent plasticity that may enable invasion through diverse extracellular matrix (ECM) barriers [12-14]. While tumor cell migration modes have been compared to normal motile cell types such as fibroblasts or immune cells [12-14], transformation to an aggressive tumorigenic phenotype profoundly disrupts signaling pathways [1-3] and cellular properties that mediate motility [15,16], including cytoskeletal organization [17-22], reorganized vinculin-containing adhesions [22-26], perturbed integrin function [27-30], and decreased adhesiveness [26,30-36]. The 3D microenvironment also plays a critical role in maintenance of normal tissue architecture [37-41], while increased proliferation, loss of tissue polarity, and transition to an invasive phenotype have been correlated to ECM influences on ERK and Rho/Rho-kinase (ROCK) signaling, cytoskeletal tension, focal adhesion structure, and integrin clustering [42-45]. Therefore, invading tumor cells migrate through mechanisms that are governed by vastly complex intracellular and extracellular signals, presenting a major challenge towards identifying therapeutic targets to treat metastatic cancers. 

Researchers have increasingly investigated tumor biology using in vitro culture platforms derived from ECM materials such as collagen or Matrigel to model the 3D microenvironment [40,41]. However, while naturally derived materials mimic the complexity of the ECM (e.g., fibrillar structure), they offer only limited control over properties that are often highly variable [46] or poorly-defined [47]. Synthetic 3D culture platforms address limitations inherent to naturally-derived materials by providing strictly defined matrix properties and have been used to investigate a wide variety of biological questions [10,48-53]. Engineered 3D models have been used to systematically investigate several questions in tumor biology [10,11,54-62], including the influence of biochemical and/or biophysical matrix properties on tumor cell migration or growth [10,11,54-56], spatiotemporal regulation of invasion by stromal cells [57], and drug response in 3D environments [54,58]. Therefore, engineering approaches complement naturally-derived culture platforms by enabling researchers to deconstruct the diverse signals of the 3D microenvironment and to systematically investigate critical factors that contribute to tumor progression [59-62].

HT-1080 fibrosarcoma cells (HT-1080s) are a human tumorigenic cell type [63-67] commonly used to model 3D tumor cell motility [5-11]. While fibrosarcoma tumors are mesenchymal in origin [68], we previously identified differences in 3D migration and morphologies for HT-1080s and human dermal fibroblasts (hDFs, a primary mesenchymal cell type [69]) that motivated the current study [10]. We hypothesized that HT-1080s were distinct from hDFs due to a malignant phenotype [65-67,70] that disrupts several signaling pathways and functional characteristics important for migration [63,64]. To test our hypothesis, we quantitatively compared adhesion properties, migration, and morphologies for HT-1080s and hDFs using defined 2D [71] and 3D [10,50] culture platforms. HT-1080s were characterized by 2D and 3D features that differed substantially from primary hDFs, but which were consistent with changes induced by transformation, including diffuse vinculin expression [22-26], decreased adhesiveness [26,30-36], and a cortically organized F-actin cytoskeleton [17-22]. Further, rounded and elongated HT-1080s and WM239a melanoma cells migrated through cortical contractility-driven mechanisms and expressed prominent uropod-like structures that were similar to features previously reported for melanoma and breast carcinoma cell lines [72-76]. Therefore, while HT-1080s were characterized by a phenotype that differed from hDFs in 2D and 3D culture, we identified distinct features that were similar to aggressive tumorigenic cell types with diverse tissue origins. Based on our results, we propose that 3D migration mechanisms for aggressive tumorigenic cell types are distinct from primary cells due to cellular changes induced by transformation.

## Materials and Methods

### Cell culture

HT-1080s (ATCC, Manassas, VA) were cultured in Alpha MEM (Lonza) supplemented with 10% fetal bovine serum (FBS, Gibco) and 1% penicillin/streptomycin (Gibco). For 2D experiments on self-assembled monolayers, primary human dermal fibroblasts (hDFs) were purchased from ATCC and cultured in Alpha MEM (Mediatech, Manassas, VA) containing 10% FBS (Invitrogen) and 1% penicillin/streptomycin (Penn/Strep). Primary hDFs used for 3D studies were kindly provided by Professor R. Rivkah Isseroff [77], Department of Dermatology, University of California-Davis and cultured in Dubellco’s Modified Eagle’s Medium (DMEM, Gibco) supplemented with 10% FBS, 1% Penn/Strep, and 0.2% fungizone. WM239a melanoma cells [78] were cultured without antibiotics in RPMI supplemented with 10% FBS. Plasmids expressing MCAM-GFP were previously described [79]. For DNA transfection, cells were trypsinized, resuspended in growth medium (WM239a cells, RPMI + 10% FBS; HT-1080s Alpha MEM + 10% FBS), washed once and then resuspended in OptiMEM. Typically, 100 μL of transfection complex (1 μg DNA, 10 μL Fugene, 100 μL OptiMEM) was added to 6 well plates (8 x10^4^ cells / 2 mL) and incubated overnight at 37°C. After overnight incubation, the media was replaced with fresh growth medium. 

### Synthetic ECM preparation

**Figure 1:**
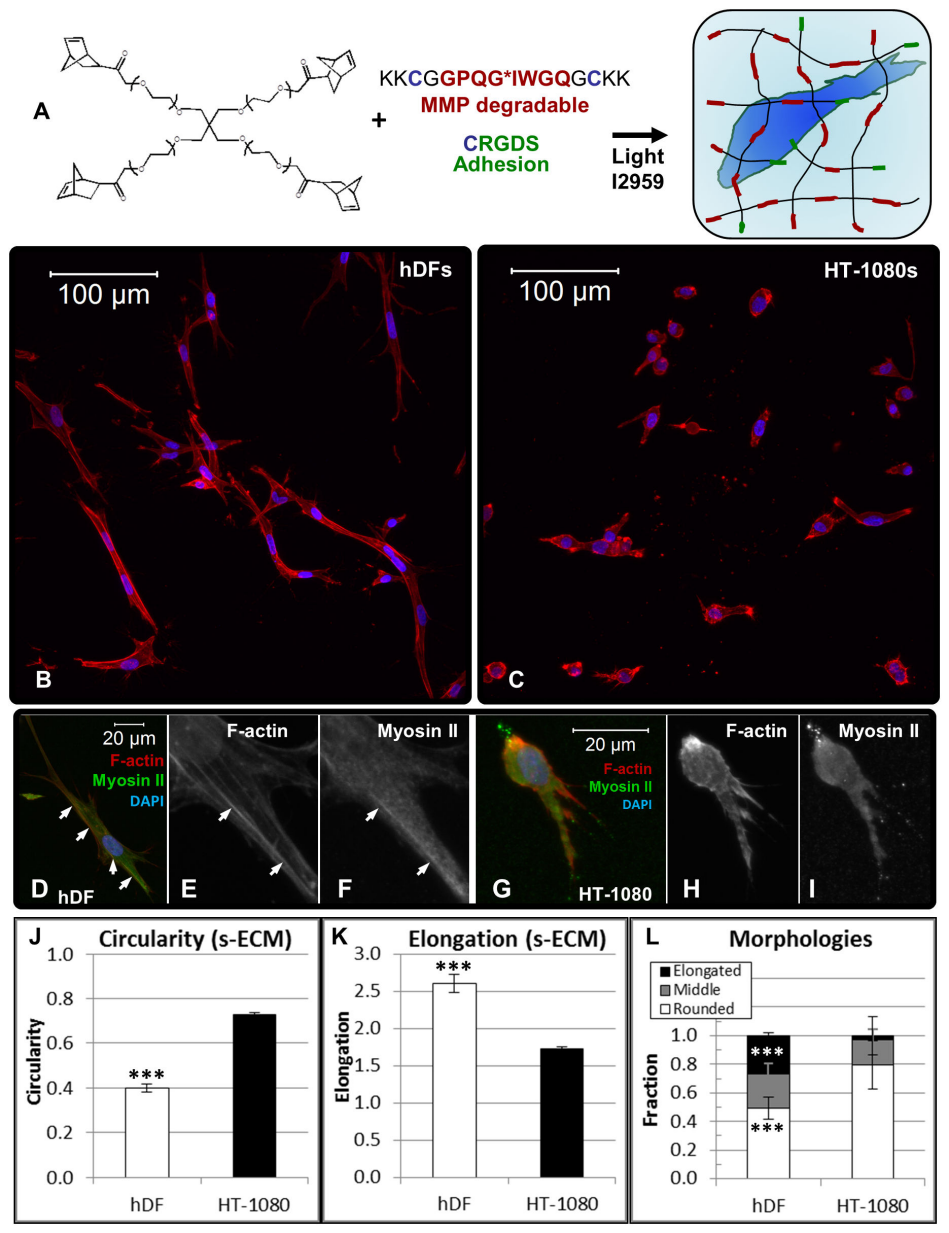
Morphologies and cytoskeletal structure for HT-1080 fibrosarcoma cells (HT-1080s) and primary human dermal fibroblasts (hDFs) in synthetic extracellular matrix (ECM). (**A**) Schematic representation of synthetic extracellular matrix (synthetic ECM) formed through “thiol-ene” photopolymerization chemistry to couple norbornene C=C bonds on 4-arm poly(ethylene glycol) (PEG) molecules with thiol (-SH) bonds of cysteine-containing peptides. Crosslinks were formed using matrix metalloproteinase (MMP)-degradable peptides with cysteine groups on each end while adhesion was promoted using pendant RGD-containing peptides (C**RGDS**) with a single cysteine (2.5 or 3 wt% by mass PEG-NB + MMP-degradable crosslinking peptide, shear moduli = 140 Pa or 220 Pa respectively). Constant total pendant peptide was maintained using non-bioactive C***RDGS*** (1500 μM active C**RGDS** + non-active C***RDGS***). (**B**-**I**) Projected z-stack immunofluorescence (IF) images illustrating hDFs and HT-1080s seeded in synthetic ECM (220 Pa, 1000 μM CRGDS). All overlay images are counterstained with TRITC-conjugated phalloidin (F-actin, red) and DAPI (nuclei, blue). (**B**,**C**) Overview to illustrate morphological differences (F-actin, red; Nuclei, blue) for (**B**) hDFs and (**C**) HT-1080s. (**D**-**F**) IF images illustrating myosin IIb expression for hDFs: (**D**) Overlay image (Myosin IIb, green; F-actin, red; Nuclei, blue); Single channel images (grayscale) illustrate (**E**) F-actin and (**F**) Myosin IIb. White arrows point to actomyosin filaments. (**G**-**I**) IF images illustrating myosin IIb expression for ***HT-1080s***: (**G**) Overlay (Myosin IIb, green; F-actin, red; Nuclei, blue); Single channel images (grayscale) illustrate (**H**) F-actin and (**I**) Myosin IIb. (**J**-**L**) Comparison of quantified mean (**J**) circularity and (**K**) elongation for hDFs and HT-1080s calculated using Nikon NIS Elements software (n > 150 cells, ≥ 6 hydrogels, at least two separate experiments; *** = p<0.001). (**L**) Fraction of elongated (Elongation ≥ 3.0), middle (2.0 ≤ Elongation < 3.0), and rounded (Elongation < 2.0) cells. Differences in fraction of elongated and rounded hDFs compared to HT-1080s were each statistically significant (N ≥ 6 total gels, at least two separate experiments; *** = p<0.001).

For 3D culture, synthetic extracellular matrix (synthetic ECM) was formed through photoinitiated coupling of thiol groups and alkenes (a “thiol-ene” reaction [80,81]) to incorporate cysteine-containing peptides into a poly(ethylene glycol) (PEG) matrix (Figure 1A) [50]. Synthetic ECM crosslinks were formed with a matrix metalloproteinase (MMP)-degradable peptide that was modified from an amino acid sequence found in collagen [82,83], while pendant peptides presenting the fibronectin-derived amino acid sequence RGD (Arg-Gly-Asp) were incorporated into the matrix for adhesion [84,85]. The RGD amino acid sequence was chosen as an adhesion ligand (in both 2D and 3D) since it binds several integrins important for motility [84,85]. Synthetic ECM formed using thiol-ene chemistry provides a well-defined 3D culture platform that is flexible towards incorporation of any biomolecule containing a thiol group and has previously been shown to be biocompatible with a variety of cell types [10,50-53]. 

Cell migration experiments using synthetic extracellular matrix (synthetic ECM) and details associated with synthesis of 4-arm poly(ethylene glycol) (PEG)-norbornene monomer and peptides were previously described in detail [10,50]. Briefly, synthetic ECM monomer solutions were prepared in PBS as a 2.5 wt% or 3 wt. % (all wt% values by mass, wt/wt) solution of 4-arm PEG-norbornene (MW 20,000) + MMP-degradable peptide cross-linker (MMP-degradable amino acid sequence: KKCGGPQG*IWGQGCKK, active sequence in bold [82,83]). 2.5 wt% and 3.0 wt% hydrogels corresponded to 140 and 220 Pa shear modulus, respectively (measured as previously described [50]). The amino acid sequence C**RGDS** (active sequence in bold) was incorporated for adhesion [84,85] and the scrambled non-bioactive amino acid sequence C*RDGS* (mutant sequence italicized) was added to maintain a constant total pendant peptide concentration of 1.5 mM (pendant peptides not included in wt% calculation). Finally, 0.05 wt% final concentration Irgacure 2959 (Ciba) was included as a photoinitiator. To encapsulate cells, a cell pellet was suspended in monomer solution at 200,000 cells/mL, and 30 μL aliquots were added to the cut end of a 1 mL syringe tip (BD Biosciences 1 mL insulin syringe, 29 G x ½ in.) and polymerized under ~10 mW cm^-2^, ~352 nm centered UV light (XX series UVP lamp) for 3 minutes. The polymerized hydrogels were then suspended in appropriate media and allowed to swell overnight before beginning experiments. 

### Inhibitors

Cell migration is limited to proteolytic mechanisms in synthetic ECM due to a small mesh size (10s of nm) [10,50]. We verified that synthetic ECM prevented non-proteolytic HT-1080 migration by exchanging normal culture media used for swelling overnight with media containing 10 μM GM6001 protease inhibitor (Millipore, 364206-1MG) (Movie S1) or equal volume of DMSO (control) for the duration of the experiment. Similar results were observed using Marimastat (Millipore, 444289-5mg). For myosin II or ROCK inhibition experiments, we added media containing 10 μM Y-27632 (ROCK inhibitor in PBS) or blebbistatin (10-50 μM, with equal volume DMSO for control) after swelling, and exposed cells to inhibitor solutions for the duration of the experiment. Time-lapse imaging began 2 hours after adding inhibitors. 

### Collagen preparation

Collagen was prepared for migration experiments at a final density of 1.7 - 3.5 mg/mL (quantified motility for 1.7 mg/mL density) using a 1:1 mixture of high concentration type I rat tail collagen (BD Biosciences, HC Collagen, 354249) and 100 mM HEPES buffer in 2X PBS, with 1X PBS or serum free AMEM added to dilute to the final density (dependent on starting concentration provided by manufacturer). A cell pellet was re-suspended at a final concentration of 200,000 cells / mL and 200 μL of the cell/collagen mixture was added to the bottom of a 48-well plate (BD Biosciences). The collagen gels were formed by incubating (37° C, 5% CO_2_) for 60 minutes, after which serum-containing media was added and cell/collagen constructs were allowed to incubate overnight before beginning time-lapse imaging. 

### Self-assembled monolayer arrays

We quantified adhesiveness for hDFs and HT-1080s using self-assembled monolayer (SAM) arrays [86] prepared as previously described in detail for other adherent cell types, without any changes in procedure [71]. Briefly, adhesion was promoted using the active RGD-containing peptide GWGG**RGDSP** (RGD, active sequence in bold) and GWGG*RGESP* (RGE, mutant italicized), where replacement of aspartic acid (D) with glutamic acid (E) provides a non-bioactive peptide to balance the total peptide density on each spot (5% total RGD + RGE). HT-1080s and hDFs were seeded onto SAM arrays (~1500 cells / cm^2^; ~32,000 cells / 60 mm dish) formed with individual RGD-functionalized spots surrounded by non-adhesive poly(ethylene glycol)-terminated regions. The size of each spot was 1.1 mm (diameter), and the maximum total cell density at 5% RGD for hDFs was ~30 cells/spot. Cells were allowed to attach for ~ 1 hr in a humidified incubator at 37 °C and 5% CO_2_, slowly dipped in warm media (in a 50 mL conical vial) to remove loosely attached cells, and then submerged in warm media contained in a rectangular multidish (Thermo Scientific/Nunc, Rochester, NY). Cell attachment was determined by the average number of cells per RGD spot (2 arrays, 7 total spots / RGD density on each array, total N = 14 spots / RGD condition). Minimum RGD density for attachment was based on a statistical increase relative to the 0 RGD (5% RGE) control condition. SAM arrays enabled efficient determination of relative adhesiveness for HT-1080s and hDFs since RGD densities spanning over three orders of magnitude were simultaneously investigated (0.002%-5% RGD mol fraction) on a single chip [71]. 

### Immunofluorescence (IF) imaging

F-actin was fluorescently tagged using TRITC-conjugated phalloidin (Sigma or Millipore. 1:100 dilution) and nuclei were stained using DAPI (either from focal adhesion kit at 1:500 dilution, Millipore, or in Prolong Gold Antifade mounting solution as received). Antibodies used were as follows: Mouse anti-vinculin monoclonal antibody (Clone 7F9, 1:100 dilution), which was included as part of a focal adhesion staining kit (Millipore, FAK100), mouse anti-integrin beta 1 monoclonal antibody (Clone 4B7R, Abcam ab3167, 1:20 dilution), rabbit anti-Myosin IIB non-muscle antibody (Sigma, M7939-.2ML, 1:100-1:500 dilution), mouse anti-MCAM (P1H12, Santa-Cruz Biotechnology), mouse monoclonal anti-Cdc2 (Santa Cruz Biotechnoloty, sc-54, 1:100 dilution), and mouse monoclonal anti-MMP14 (Abcam, ab56307, 1:100 dilution). Secondary antibodies used were either Alexa-Fluor 488 goat anti-rabbit (Invitrogen, A-11008) or goat anti-mouse (Invitrogen, A-11001) IgG (both used at 1:200 dilution). 

 IF staining followed the procedure provided for a focal adhesion staining kit (Millipore, FAK100). For 3D IF staining, we modified the procedure by expanding incubation times to account for diffusion within the poly(ethylene glycol) (PEG) hydrogels used to form our synthetic ECM. Cells within synthetic ECM hydrogels were fixed using 4% formaldehyde in PBS for 30 minutes. Following fixing, arrays were washed 3 times with wash buffer (0.05% Tween 20, 3 x 15 minute incubation for each rinse). Next, cells were permeabilized using 0.1 % Triton X-100 in PBS for 30 minutes, followed by additional rinsing with wash buffer (3 x 15 min.). Gels were then blocked using 1% (w/w) bovine serum albumin (BSA, Fisher Scientific) for 2 hours. Primary antibody solutions were then prepared in blocking solution (1% BSA), added to gels, and incubated overnight at 4° C. After rinsing with wash buffer (3 x 30 minutes, extra time to ensure all primary antibodies were removed), a solution prepared in PBS containing secondary antibody, TRITC-conjugated phalloidin, and DAPI was applied overnight at 4° C. The final rinse was in PBS rather than wash buffer (3 x 30 minutes, gels were left in PBS at 4° C until use, typically within 48 hours of preparation). For some myosin II images, cells within synthetic ECM were fixed with 2% buffered paraformaldehyde (30 minutes), blocked and permeabilized using 0.25% Triton X-100 and 1% BSA in PBS (60 minutes), incubated with primary myosin II antibody (1:500, 0.05% Triton X and 1% BSA in PBS; 4° C overnight), washed three times (2 x 15 min., final rinse overnight at 4° C, 0.05% Triton X in PBS), incubated with secondary antibody (1:200 antibody, 1:500 DAPI and 1:500 phalloidin, 0.05% Triton X and 1% BSA in PBS; 4° C, 4 hours), and thoroughly washed to allow excess antibodies to diffuse from hydrogel (1 hr. 0.05% Triton X in PBS., overnight in PBS). 

For IF imaging in synthetic ECM, gels were placed on a glass slide and surrounded by a 1 mm silicone rubber gasket (McMaster-Carr, with a hole formed using a 5 mm tissue punch). The thickness of the hydrogel was slightly higher than the gasket, creating a tight seal when a coverslip was placed on top (the gasket prevented the gel from being flattened). Most IF images in synthetic ECM were collected in 8-bit multi-track mode (Wavelengths: 488 nm on one track, 405 and 543 nm on the other) with an LSM 710 (Zeiss) confocal microscope using a 40X W Plan-Apochromat objective (1.0 DIC M27) or a 20X W Plan-Apochromat objective (1.0 DIC M27). Some IF images for myosin II were collected using a Nikon A1R confocal microscope (courtesy of Prof. Randolph Ashton, University of Wisconsin-Madison). All 3D images were displayed as flattened z-projections (Zeiss Image Browser, maximum transparency; NIS elements, maximum intensity projection) unless otherwise noted. Intensity profiles were generated using the ImageJ “Plot Profile” function, while false-color intensity images were generated with the “Interactive 3D Surface Plot” plugin [87-89] or using the LSM Image Browser. 

### Quantifying migration and morphologies

Quantified 3D migration in synthetic ECM was determined using an automated Nikon TE2000-E inverted microscope, controlled with Metamorph software, and a Nikon environmental chamber with external heater (In vivo Scientific) and CO_2_ regulator (In vivo Scientific) to control temperature (37° C) and CO_2_ (5%) levels. Quantified attachment to self-assembled monolayers and migration in collagen gels were determined using an automated Nikon Ti-E inverted microscope, controlled with NIS Elements (Nikon) software, and a Tokai Hit stage top incubation system to control temperature (37° C) and CO_2_ (5%) levels. 

Time-lapse microscopy was used to track cells in synthetic ECM for 6 hours (15 minute increments) and analyzed for quantified migration and morphologies using MetaMorph or NIS-Elements (Nikon) software. Quantified migration was determined by tracking cells using 2D minimum intensity projections of z-stacks (10 μm slices, middle 500 μm of ~1000 μm thick hydrogels). The position of a non-cellular feature within the hydrogel was tracked and then subtracted from each cell position to correct for drift. A migrating cell in synthetic ECM and collagen was defined as having traveled a distance greater than one cell length from the starting position anywhere on the 2D projected cell track during the 6 hours of analysis. One cell length was defined based on the value determined for hDFs using morphology analysis (NIS Elements) in our base synthetic ECM formulation (220 Pa, 1000 μM CRGDS, see Figure 1H) and was applied for all 3D migration analysis. Cells that interacted or divided were not included in analysis. The same 6 hour time course was used for control cells and cells treated with inhibitors. Imaging began 2 hours after adding fresh media with or without inhibitors (and after overnight swelling in control media). Reported values for experiments in synthetic ECM represent a 3D correction for 2D minimum intensity z-projections used for tracking (multiplied by a factor of √3/2, migration was assumed to be independent of x,y, or z direction). Directionality (DTO/TD, Distanct-to-origin / Total distance) is a dimensionless parameter that provides a measure of directional motility analogous to persistence time [90] (Figure S1A). 

NIS Elements was used to quantify morphologies from the outline for the best focus of each cell using the “Draw Object” feature, from which circularity, elongation, and other measurements reported here were calculated with the “Automated Measurement” feature. Circularity is a function of area and perimeter (Circularity = 4π*area/perimter^2^) that is maximum for a circle (Circularity = 1). Elongation represents the ratio of MaxFeret / MinFeret defined by NIS Elements (approximately the ratio of the long axis to the short axis of the cell), and was used to further classify cells into categories defined as rounded (EI < 2.0), middle (2.0 ≤ EI < 3.0), and elongated (EI ≥ 3.0), similar to a method previously described for HT-1080s in collagen [6]. Some movies and time-lapse images used for display were corrected for drift using the StackReg plugin (“Translation” function) [91] for ImageJ [87,88]. All statistical analysis was performed using a two-tailed student’s t-test. All error bars represent standard error of the mean (SEM) unless otherwise noted.

## Results

### HT-1080 fibrosarcoma cells (HT-1080s) and human dermal fibroblasts (hDFs) have distinct phenotypes in 2D and 3D culture

We first investigated cytoskeletal organization and quantified morphologies due to the distinct rounded appearance for HT-1080s compared to hDFs in 3D culture (Figure 1, Figure S2). In synthetic ECM, hDFs were characterized by cytoskeletal properties that were similar to primary fibroblasts in naturally-derived [92-97] and synthetic [98] ECM materials, including elongated, multipolar morphologies and F-actin dense protrusions (Figure 1B). Further, hDFs formed organized actomyosin filaments with co-localized F-actin and myosin II (Figure 1D-F, arrows) in synthetic ECM, which was consistent with previously studies using a variety of 3D culture platforms [92-98]. In contrast, HT-1080s adopted rounded or spindle shaped morphologies (Figure 1C) characterized by a cortically organized F-actin cytoskeleton and myosin II that was diffusely expressed throughout the cytoplasm (Figure 1G-I). A quantitative comparison of morphologies (see Methods) demonstrated that HT-1080s were characterized by increased circularity (Figure 1J), decreased elongation (Figure 1K), a higher fraction of rounded cells (Figure 1L), a lower fraction of elongated cells (Figure 1L), and a shorter average cell length (Figure S1D) than hDFs. Thus, HT-1080s were characterized by distinct cytoskeletal structure and a quantitatively more rounded phenotype than hDFs. 

**Figure 2:**
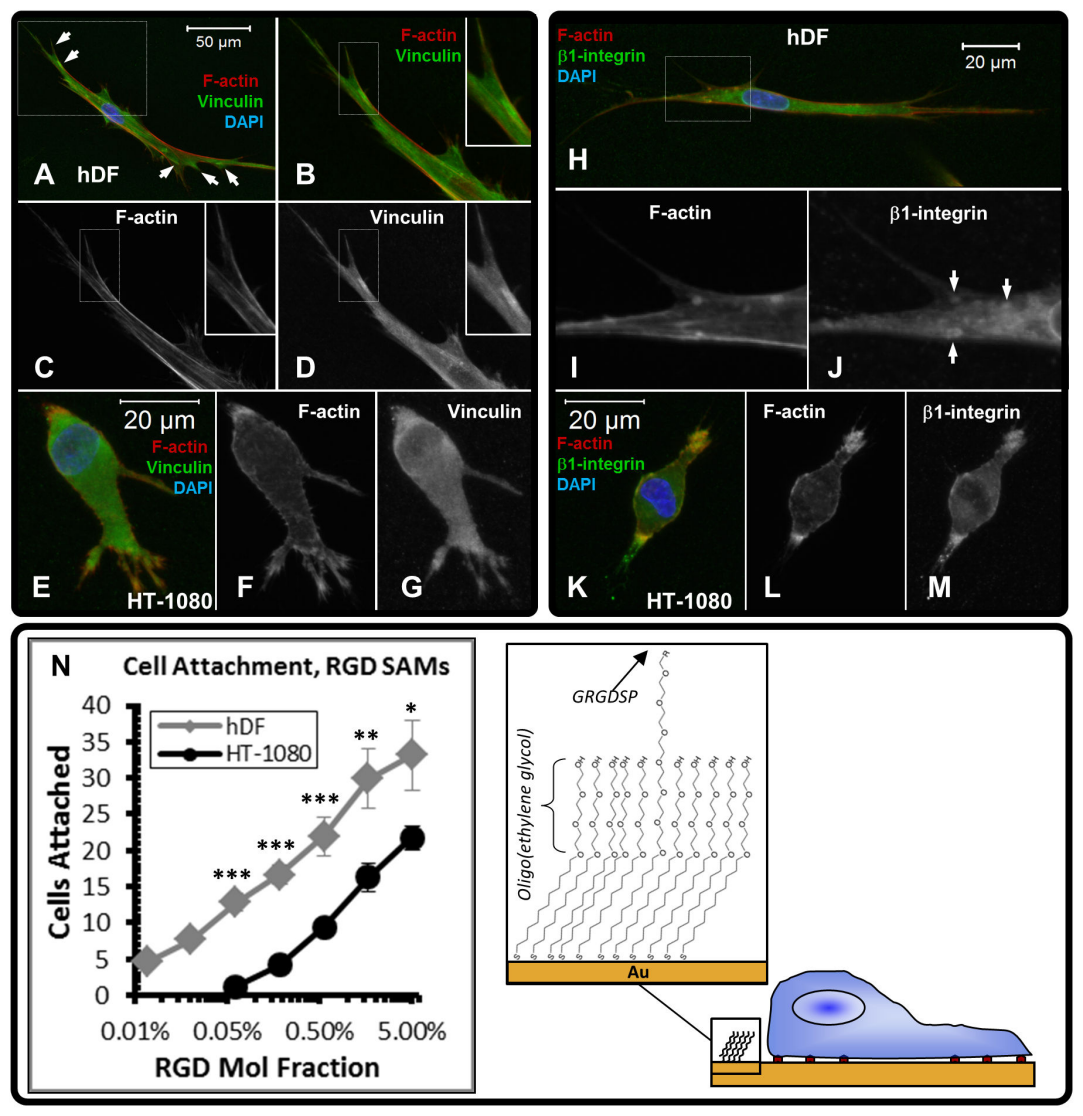
Comparison of adhesion properties for HT-1080s and hDFs. (**A**-**M**) Projected z-stack immunofluorescence (IF) images for hDFs and HT-1080s in synthetic ECM (220 Pa, 1000 μM CRGDS). All overlay images are counterstained with TRITC-conjugated phalloidin (F-actin, red) and DAPI (nuclei, blue). (**A**-**D**) IF images illustrating vinculin expression for ***hDFs*** (boxed region from A shown in B-D): (**A**) Overlay image (Vinculin, green; F-actin, red; Nuclei, blue). White arrows point to regions enriched with vinculin. (**B**) Overlay (Vinculin, green; F-actin, red); Single channel images (grayscale) illustrate (**C**) F-actin and (**D**) Vinculin. (**E**-**G**) IF images illustrating vinculin expression for ***HT-1080s***: (**E**) Overlay image (Vinculin, green; F-actin, red; Nuclei, blue). Single channel images (grayscale) illustrate (**F**) F-actin and (**G**) Vinculin. (**H**-**J**) IF images illustrating β1-integrin expression for ***hDFs***: (**H**) Overlay (β1-integrin, green; F-actin, red; Nuclei, blue); Single channel images (grayscale) illustrate (**I**) F-actin and (**J**) β1-integrin (White arrows point to punctate β1-integrin features; Also Fig. S3). (**K**-**M**) IF images illustrating β1-integrin expression for ***HT-1080s***: (**K**) Overlay (β1-integrin, green; F-actin, red; Nuclei, blue); Single channel images (grayscale) illustrate (**L**) F-actin and (**M**) β1-integrin. (**N**) Comparison of attachment for hDFs and HT-1080s on RGD-SAMs as a function of RGD density. Attachment was statistically significant (cell number > 0 RGD control spots) for HT-1080s on surfaces with ≥ 0.19% mol fraction RGD and for hDFs on surfaces ≥ 0.007% mol fraction RGD. Error bars represent standard error of the mean (SEM) for array spots at given RGD density. Significance for cell attachment on individual spots was calculated for hDFs relative to HT-1080s (* = p<0.05; ** = p<0.01; *** = p<0.001).

HT-1080s also had distinct adhesion characteristics compared to hDFs (Figure 2). hDFs were characterized by vinculin-rich features on the tips of protrusions (Figure 2A) and β1-integrin spots throughout the cell (Figure 2H, Figure S3) in synthetic ECM, which resembled adhesion structures previously described for fibroblasts in 3D culture [94,95,99]. HT-1080s expressed vinculin diffusely throughout the cytoplasm in synthetic ECM (Figure 2E), which was consistent with reported expression in collagen [9], but contrasted with the defined features observed for hDFs. HT-1080s expressed β1-integrin as condensed features on the tips of protrusions and on rear end uropod-like structures (Figure 2K, Figures S4-S5), which was also distinct from adhesions observed for hDFs. Next, we compared attachment of HT-1080s and hDFs to RGD-functionalized self-assembled monolayers (RGD-SAMs, Figure 2N) to provide a quantitative measure of adhesiveness. Fewer HT-1080s attached to RGD-SAMs than hDFs for all RGD densities investigated. HT-1080s also required >25-fold higher RGD density than hDFs to attach to RGD-SAMs (0.19% RGD for HT-1080s, 0.007% RGD for hDFs, see Methods). Results for hDF adhesion here were similar to human mesenchymal stem cells (hMSCs) and human umbilical vein endothelial cells (HUVECs) that we previously compared using identical RGD-SAM array formulations [71]. Therefore, HT-1080s were less adhesive than several primary human cell types and were characterized by distinct adhesion properties compared to hDFs in both 2D and 3D environments. 

**Figure 3:**
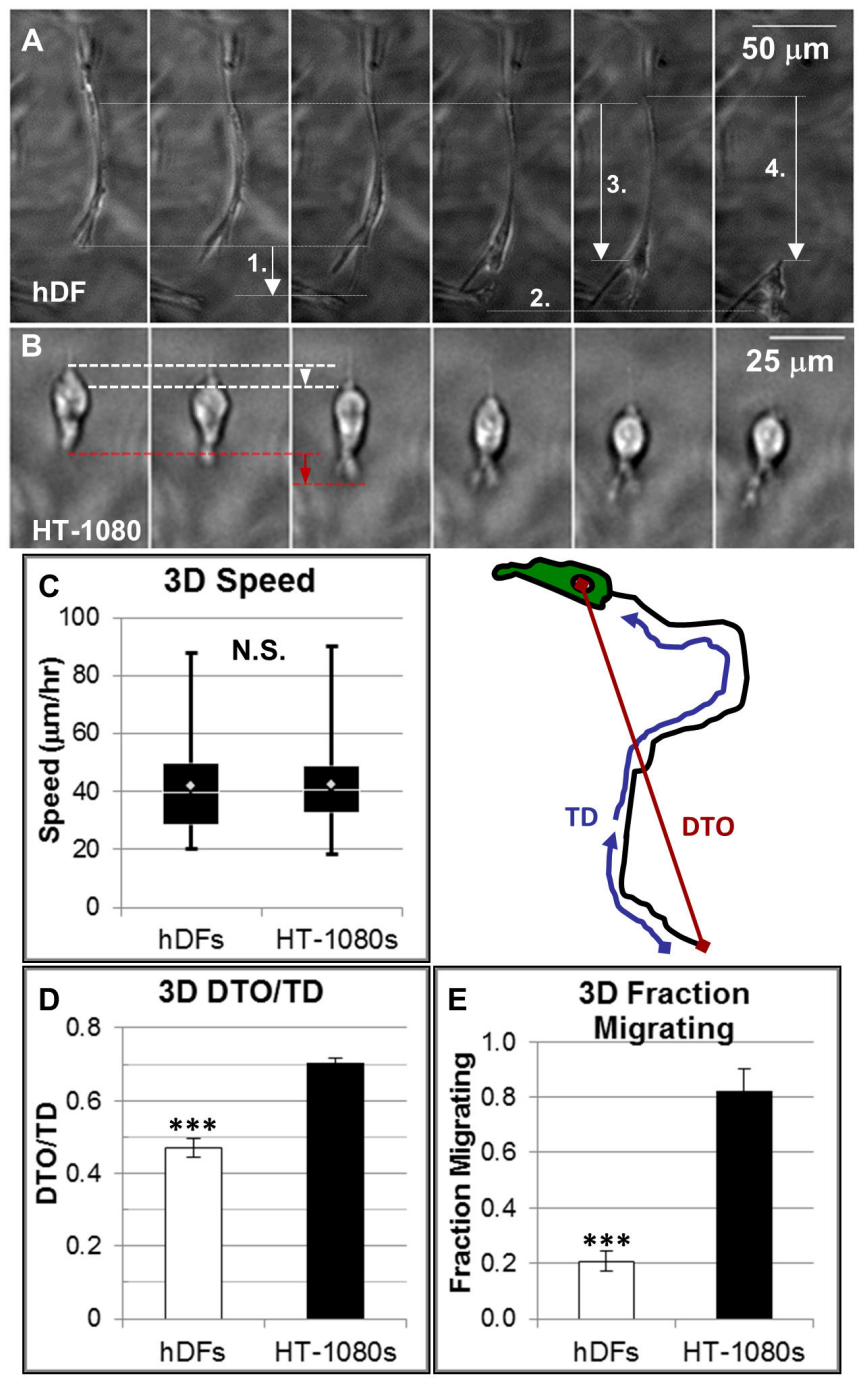
Qualitative and quantitative migration for HT-1080s and hDFs. Time-lapse images (10 min./frame) illustrating migration for (**A**) an hDF and (**B**) an HT-1080 in synthetic ECM (220 Pa, 1000 μM CRGDS). Image sequences in (**A**,**B**) are contrast and brightness enhanced for better display (see Movies S2, S3 for unaltered images, Movie S4 for overview comparison of many cells). (**A**) Migration movement for hDF is characterized by: 1. Front end extension, 2. Attachment, 3. Cell body contraction, and 4. Rear-end release. (**B**) HT-1080 movement illustrates cell body contraction (white dashed lines and arrow) and simultaneous front end extension (red dashed lines and arrow). (**C**-**E**) Comparison of quantified 3D migration for HT-1080s and hDFs in synthetic ECM (220 Pa and 1000 μM CRGDS): (**C**) Cell speed (adjusted by a factor of √3/2, which was a 3D correction for analysis on 2D minimum intensity z-projections), (**D**) directionality (DTO/TD), and (**E**) fraction migrating cells. DTO/TD is a dimensionless parameter that provides a measure of directional motility analogous to persistence time (Fig. S1A) that is calculated as the distance-to-origin (DTO) after 6 hours divided by the total path length (total distance, TD) (shown schematically, panel to right of C). Migration was calculated from images collected in 15 min. increments for 6 hours, with dividing or interacting cells excluded (≥200 cells, ≥ 3 separate experiments, ≥ 9 total hydrogels). *Box* and *whisker* *plot* *for* *cell* *speed*: White diamond = mean, white line = median, boxes = middle upper (top) and middle lower (bottom) quartile of the cell population, whiskers = highest (above) and lowest (below) migration speeds. Values for DTO/TD represent the mean for all cells while fraction migrating represents the mean for replicate experiments (N ≥ 3). Significance was calculated for hDF relative to HT-1080 migration for each parameter (*** = p<0.001).

Finally, HT-1080s migrated with distinct movement and a more polarized morphology when compared to hDFs (Figure 3; Movies S2-S4). Whereas hDFs were characterized by elongation, attachment, cell body translocation, and rear release (Figure 3A), HT-1080s migrated with prominent cell body contraction (white dashed lines and arrows, Figure 3B) and protrusion extension that was primarily limited to the leading edge (Figure 3B, red dashed lines and arrows). A comparison of quantified migration demonstrated that cell speed was similar for HT-1080s and hDFs (Figure 3C). However, HT-1080s migrated with increased directionality (Figure 3D, Distance-to-Origin/Total Distance, “DTO/TD”), which led to a substantially higher fraction of migrating cells than hDFs (Figure 3E, Figure S1E). Collectively, our results indicated that HT-1080s were more polarized than hDFs in synthetic ECM, which was reflected qualitatively in morphologies and protrusion dynamics, and quantitatively by increased directional migration and a relatively more motile phenotype. Taken together, HT-1080s were characterized by expression of adhesion proteins, adhesiveness, cytoskeletal organization, morphologies and motility that differed substantially from hDFs.

### Rounded and elongated HT-1080s migrate through cortical contractility-driven proteolytic mechanisms in synthetic ECM

**Figure 4:**
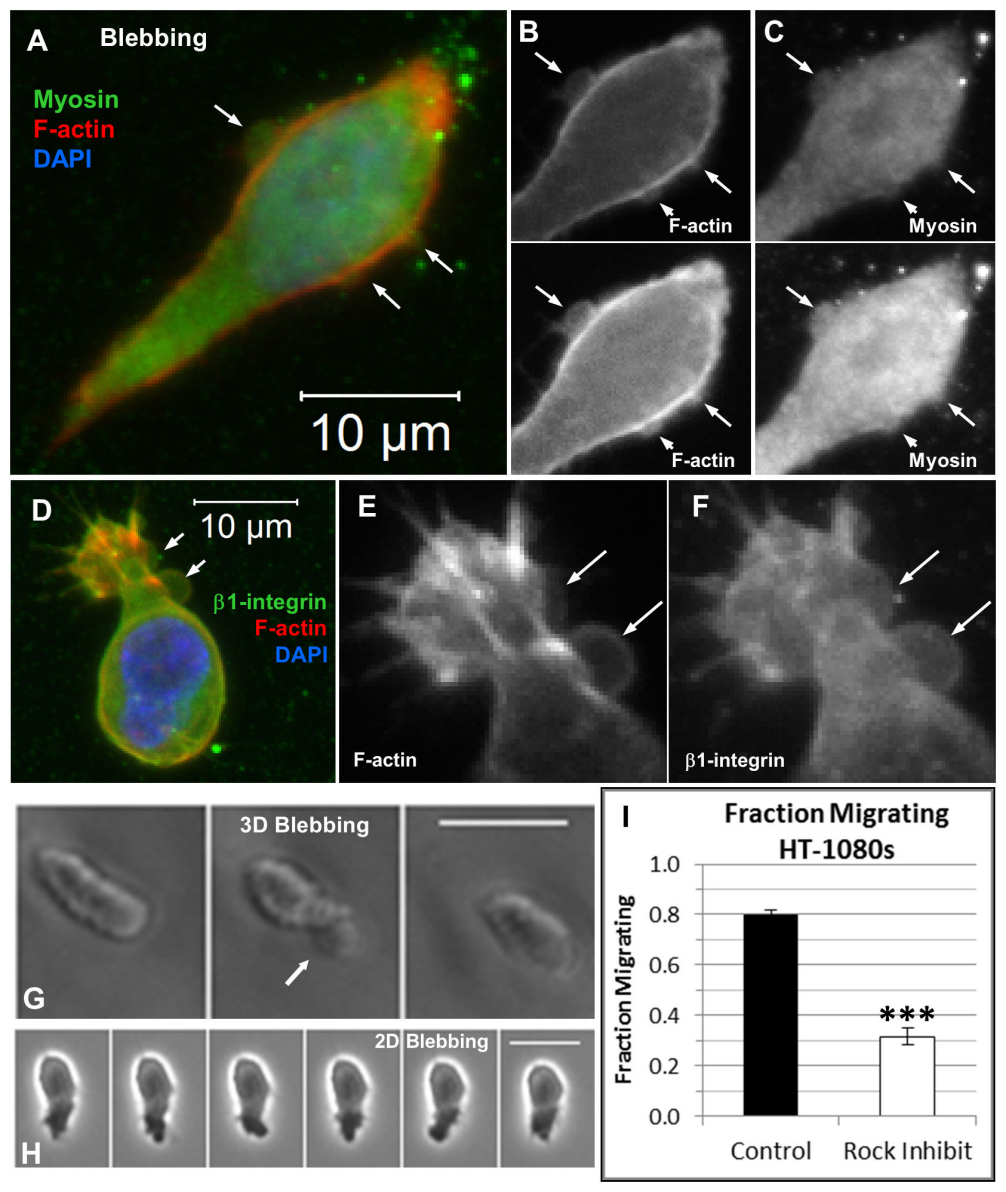
Blebbing and contractility-dependent motility for HT-1080s in 2D and 3D culture. (**A-F**) Projected z-stack immunofluorescence (IF) images for HT-1080s in synthetic ECM (220 Pa, 1000 μM CRGDS). Overlay images are counterstained with TRITC-conjugated phalloidin (F-actin, red) and DAPI (nuclei, blue). Arrows point to apparent blebs or cortex rupture. (**A**) Overlay image (Myosin IIb, green; F-actin, red; Nucleus, blue). Unprocessed (top) and brightness enhanced (bottom) single channel images (grayscale) illustrate (**B**) F-actin and (**C**) myosin IIb. (**D**) Overlay image (β1-integrin, green; F-actin, red; Nucleus, blue) for a cell migrating into the plane; Single channel images (grayscale) illustrate (**E**) F-actin and (**F**) β1-integrin. Time-lapse images illustrating apparent bleb formation (arrows) for HT-1080s migrating (**G**) in synthetic ECM (220 Pa, 1000 μM CRGDS; 10 min / frame, See also Movies S5, S6) and (**H**) on a 1.7% mol. fraction RGD-SAM surface (30 sec / frame, See also, Movie S8). (**I**) Fraction of HT-1080s migrating in synthetic ECM (220 Pa, 1000 μM CRGDS): Control (black) and treated with ROCK inhibitor (white, 10 μM Y27632). See Movie S10 for treatment with myosin II inhibitor (Blebbistatin). Significance was calculated for average fraction of cells migrating per hydrogel (11 hydrogels, 4 separate experiments; *** = p<0.001). Scale bars = 25 μm unless otherwise noted.

**Figure 5:**
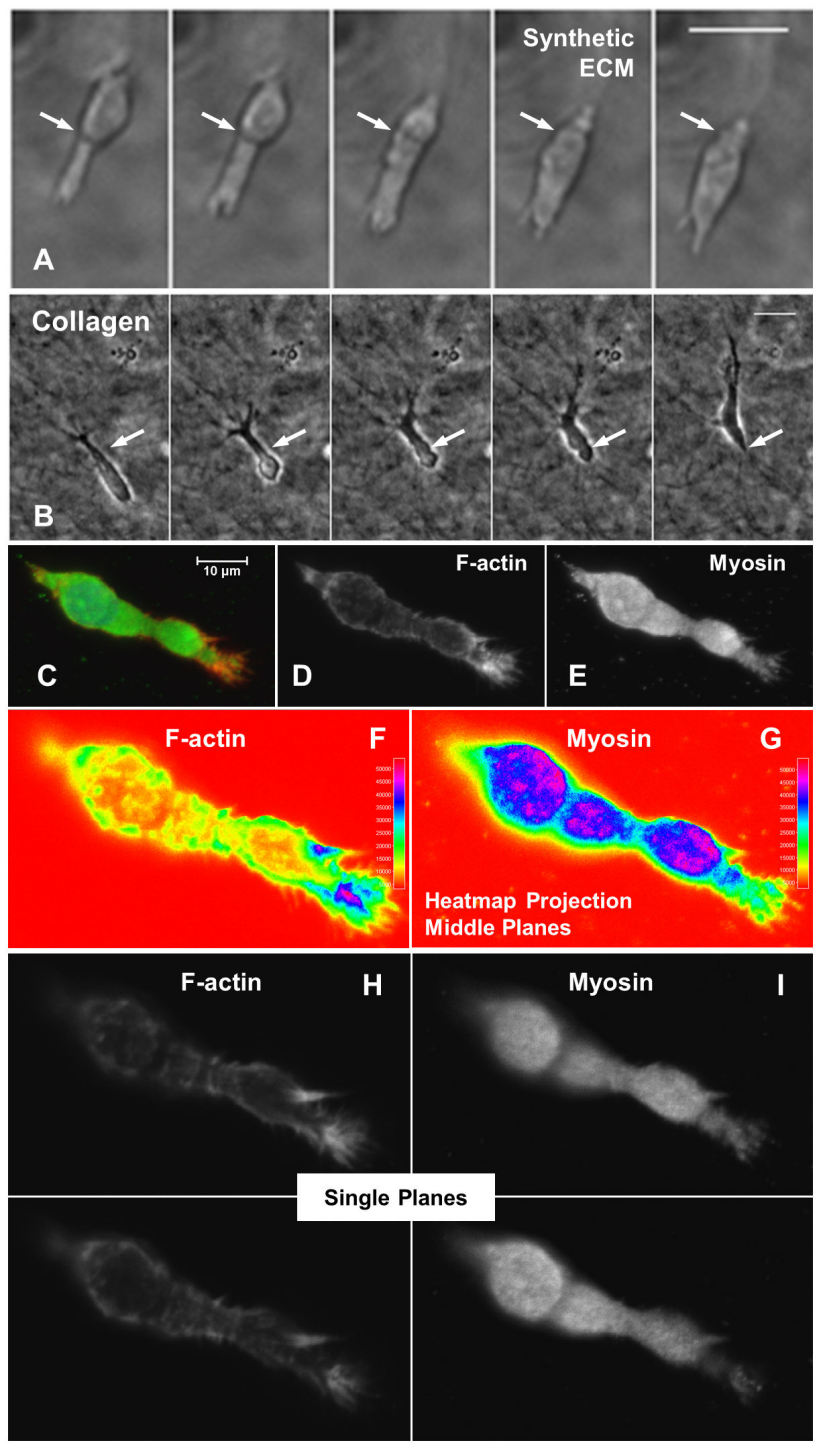
Contractile movement for HT-1080s in 3D culture. (**A**) Propagation of a constriction ring (arrow) for an HT-1080 migrating in synthetic ECM (220 Pa, 1000 μM CRGDS; 10 min / frame, see also Movie S3). (**B**) Propagation of a constriction ring (arrow) for an HT-1080 migrating in collagen (1.7 mg/mL; 15 min./frame, see also Movie S9). Scale bars = 25 μm. (**C**-**G**) Z-projected immunofluorescence images illustrating myosin IIb expression for an HT-1080 in synthetic ECM (220 Pa, 1000 μM CRGDS): (**C**) Overlay image illustrating myosin IIb (green), counterstained with TRITC-conjugated phalloidin (F-actin, red) and DAPI (nucleus, blue). Single channel images (grayscale) illustrate (**D**) F-actin and (**E**) Myosin IIb. Profile plots generated using ImageJ “Interactive 3D Surface Plot” function (“Spectrum” intensity scale, projection for middle 3 planes) illustrate (**F**) F-actin and (**G**) Myosin IIb. Two consecutive single plane images (grayscale) illustrate (**H**) F-actin and (**I**) Myosin IIb.

**Figure 6:**
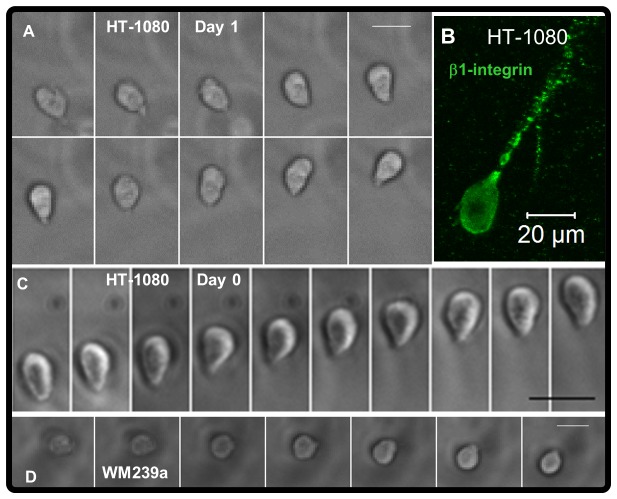
Rounded proteolytic migration modes for tumor cells in synthetic ECM. Rounded tumor cells migrating in synthetic ECM (220 Pa, 1000 μM CRGDS): (**A**) Time-lapse images (15 min / frame; See also Movie S11) for a rounded HT-1080 after overnight swelling (day 1). (**B**) β1-integrin expression (immunofluorescence) for a rounded HT-1080. (**C**) Time-lapse images (10 min / frame; See also Movie S12) for a rounded HT-1080 shortly after encapsulation (Initial migration, Day 0). (**D**) Time-lapse images (30 min / frame) for a rounded WM239a melanoma cell after overnight swelling (day 1). Scale bars = 25 μm unless otherwise noted.

**Figure 7:**
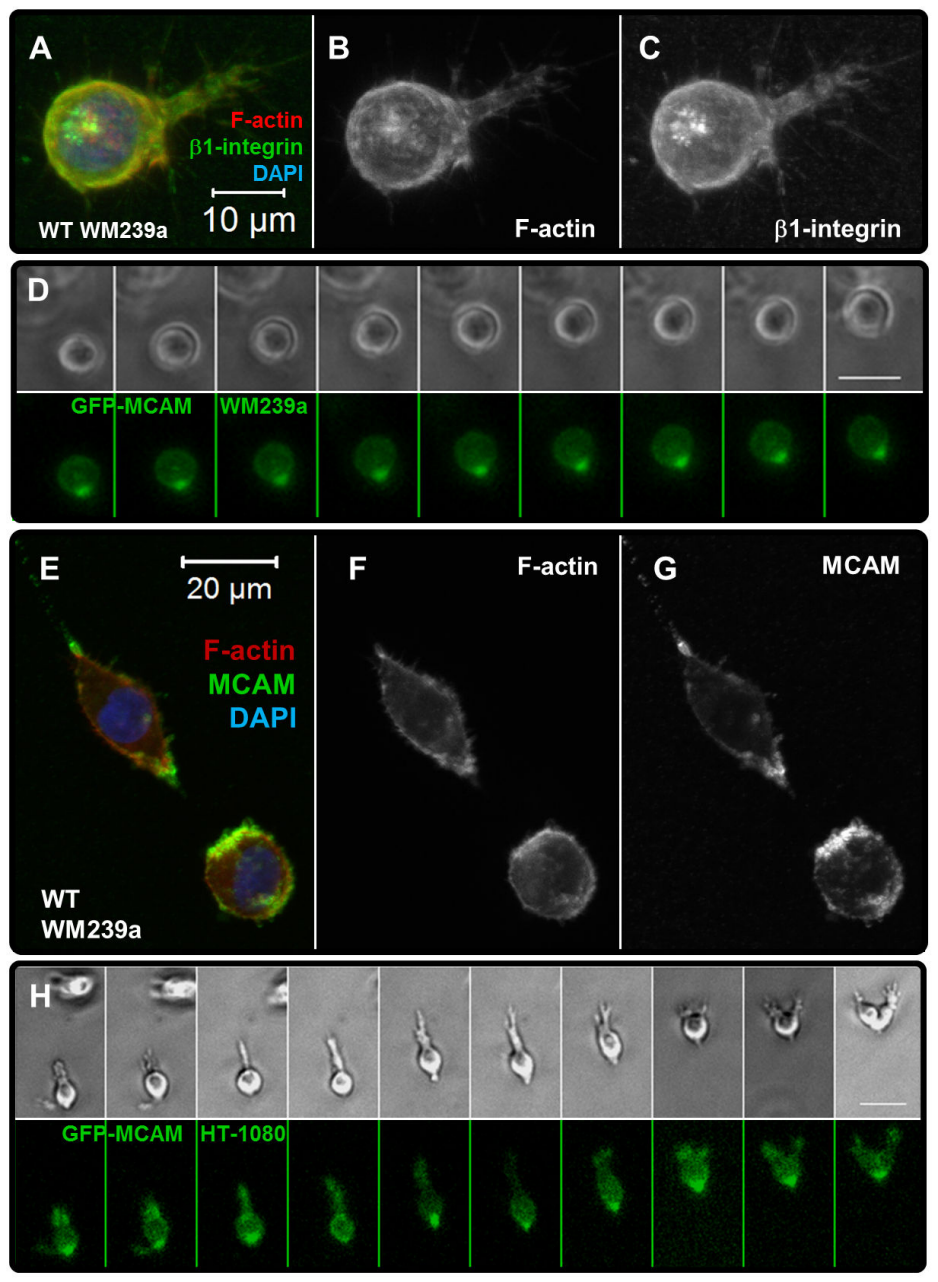
HT-1080s and WM239a cells are characterized by rear-end MCAM-expressing uropod like structures. Illustration of uropod-like features and MCAM expression for tumor cells in synthetic ECM (220 Pa, 1000 μM CRGDS): (**A**-**C**) Z-projected immunofluorescence images for a WM239a melanoma cell; (**A**) β1-integrin (green), counterstained with TRITC-conjugated phalloidin (F-actin, red) and DAPI (nucleus, blue); Single channel images (grayscale) illustrate (**B**) Factin and (**C**) β1-integrin. (**D**) Time-lapse images for a GFP-MCAM transfected WM239a melanoma cell (30 min / frame; See also Movie S13); Brightfield (top) and GFP-MCAM expression (bottom). (**E**-**G**) Z-projected immunofluorescence images for a WM239a melanoma cell; (**E**) Melanoma cell adhesion molecule (MCAM, green), counterstained with TRITC-conjugated phalloidin (F-actin, red) and DAPI (nucleus, blue); Single channel images (grayscale) illustrate (**F**) Factin and (**G**) MCAM. (**H**) Time-lapse images for a GFP-MCAM transfected HT-1080 cell (15 min / frame; Background pixel intensity subtracted using the “Math” function in ImageJ and uniformly applied to all GFP images for better display); Brightfield (top) and GFP-MCAM expression (bottom).

Contractility and hydrostatic pressure-driven processes played an important role for driving HT-1080 migration through both rounded and elongated morphologies in synthetic ECM (Figures 4-5, Figures S5-S6). Blebs or bleb-like features were prominent for HT-1080s in synthetic ECM (Figure 4, Figures S5-S6; Movies S5, S6, S7), including on the cell body (Figure 4A-C), along leading protrusions (Figure 4D-F), and at the tips of protrusions (Figure 4G). Blebs were also observed on RGD-SAMs (Figure 4H; Movie S8), suggesting that hydrostatic pressure played a role for 2D motility [100,101]. Inhibition of Rho-kinase (ROCK) reduced the fraction of HT-1080s migrating in synthetic ECM (Figure 4I), indicating that migration was contractility-dependent. HT-1080s also formed constriction rings or “cortical contraction waves” [102-104] in synthetic ECM (Figure 5A; Movie S3) and collagen (Figure 5B; Movie S9), which was consistent with mechanisms previously described for immune cell [100-107] and tumor cell [5,108] motility. Myosin II expression was elevated in bulged regions and appeared to be depleted in constrictions between them for HT-1080s in synthetic ECM (Figure 5C-I), while Blebbistatin (a myosin II inhibitor) induced a mostly non-motile phenotype (Movie S10). HT-1080s lacking visible protrusions were still highly motile in synthetic ECM (Figure 6A-C; Movies S11,S12), and similar migration modes were observed for WM239a melanoma cells (Figures 6D, 7D; Movie S13). Rounded migration modes were observed for HT-1080s in synthetic ECM immediately after encapsulation (Figure 6C; Movie S12), which confirms the requirement for proteolysis since cell tracks that would enable non-proteolytic motility had not been formed. Based on these results, we conclude that both rounded and elongated tumor cells migrate through cortical contractility-dependent proteolytic mechanisms.

HT-1080s were characterized by condensed F-actin and β1-integrin on rear-end features in synthetic ECM (Figure 2K, Figures S4-S5) that were similar to distinct uropod-like structures previously described for tumor cells migrating in 3D matrices [72-75]. Melanoma cell adhesion molecule (MCAM/cd146/MUC18) is a component of a “Wnt-Receptor-Actin-Myosin-Polarity (W-RAMP)” structure that mediates polarity and protrusion retraction [79], and was previously identified on a uropod-like structure for WM239a melanoma cells in synthetic ECM [72]. Therefore, we compared MCAM expression for HT-1080s and WM239a cells to investigate a potential role for the uropod-like structure for tumor cells migrating in synthetic ECM. As was observed for HT-1080s, WM239a cells were characterized by condensed F-actin and β1-integrin on rear-end uropod-like features in synthetic ECM (Figure 7A-C, Figure S7). GFP-MCAM transfected WM239a cells adopted rounded morphologies and migrated with the MCAM feature pinned at the rear (Figure 7D; Movie S13), which is in agreement with our previous results [72]. MCAM was also expressed on the front and rear for non-transfected WM239a cells (Figure 7E-G) and for both GFP-expressing and control HT-1080s in synthetic ECM (Figure 7H, Figure S7C-D). Thus, MCAM played a dynamic role for tumor cells migrating in synthetic ECM, and was a prominent feature on the uropod-like structure for rounded and elongated migration modes. 

### Quantified HT-1080 migration was weakly-dependent on biochemical and biophysical properties despite substantially altered morphologies

**Figure 8:**
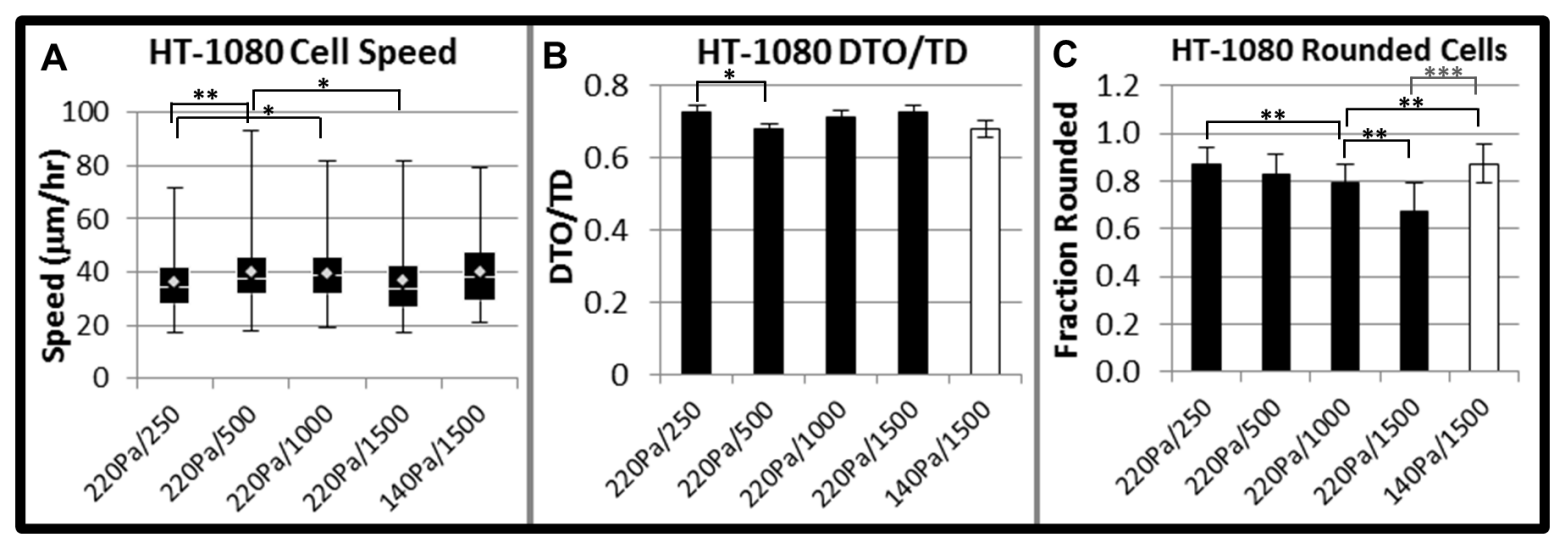
Matrix influences on migration and morphologies for HT-1080s in synthetic ECM. (**A**) Cell speed and (**B**) directionality (DTO/TD) for HT-1080s as a function of matrix conditions (≥ 6 gels, ≥ 40 cells, at least two separate experiments; * = p<0.05; ** = p<0.01). X-axis: Modulus in Pa / RGD concentration in μM. *Box* and *whisker* *plot* *for* *cell* *speed*: White diamond = mean, white line = median, boxes = middle upper (top) and middle lower (bottom) quartile of the cell population, whiskers = highest (above) and lowest (below) migration speeds. Error bars for DTO/TD represent standard error of the mean for individual cells. There is also a statistical difference in cell speed for the 140 Pa (1500 μM RGD) and 220 Pa (250 μM RGD) conditions (p<0.05, not shown on graph for clarity). (**C**) A comparison of the fraction of rounded HT-1080s (Elongation Index < 2.0) as a function of synthetic ECM conditions (x-axis: Modulus in Pa / RGD concentration in μM; white bar = 140 Pa; black bars = 220 Pa). Error bars represent standard deviation for fraction of rounded cells per gel (≥ 6 gels, at least two separate experiments; * = p<0.05; ** = p<0.01; *** = p<0.001).

**Figure 9:**
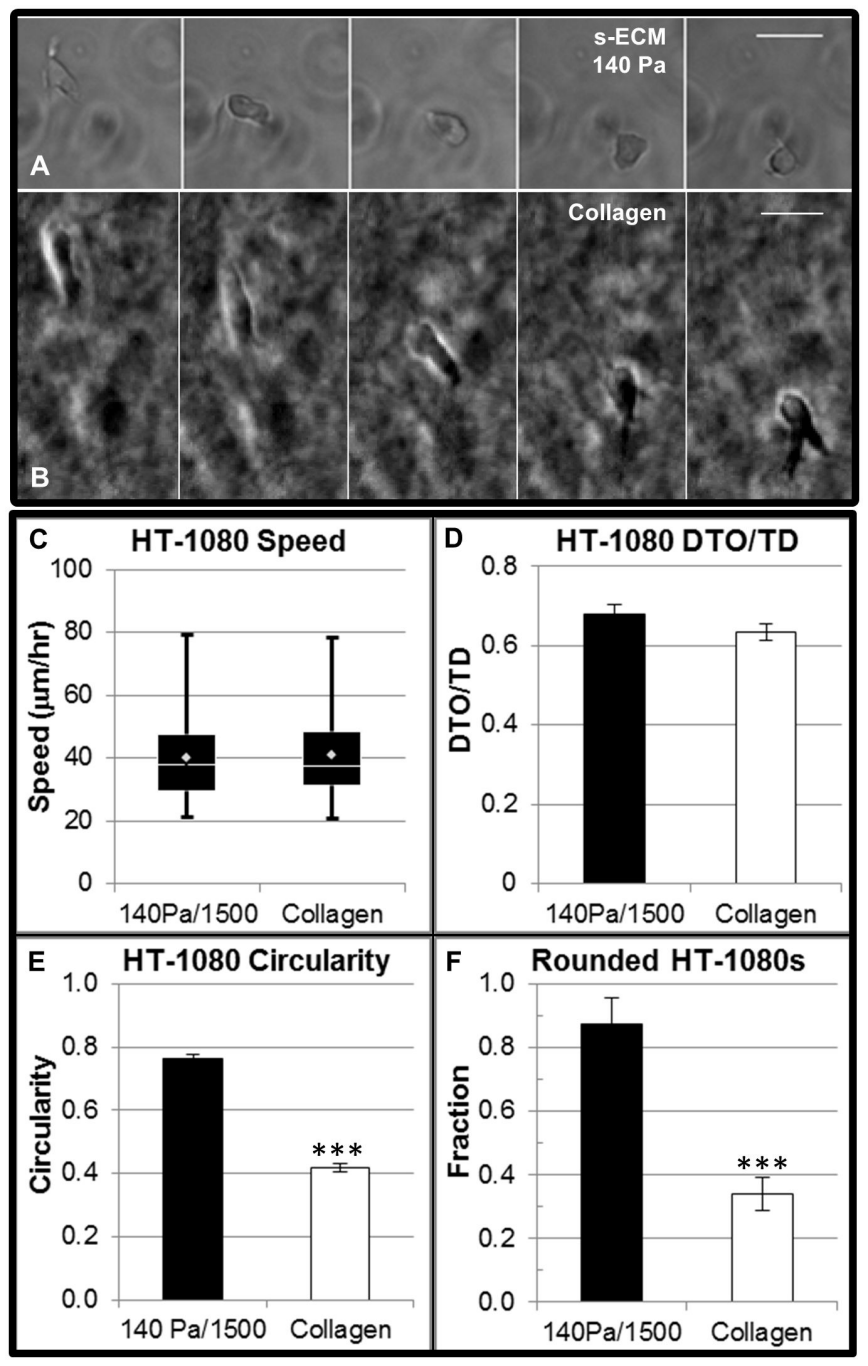
A comparison of quantified migration and morphologies for HT-1080s in synthetic ECM and collagen. Migration and morphologies were compared for HT-1080s cultured in lower modulus synthetic ECM (140 Pa, 1500 μM CRGDS) and collagen (1.7 mg/mL, acid-extracted, rat tail collagen, BD Biosciences). Time-lapse microscopy (15 min / frame) illustrating HT-1080s migrating in (**A**) synthetic ECM (also Movie S15) and (**B**) collagen (also Movie S9). Scale bars = 25 μm. HT-1080s migrated in lower modulus synthetic ECM and collagen with statistically equivalent (**C**) speed and (**D**) directionality (DTO/TD). *Box* and *whisker* *plot* *for* *cell* *speed*: White diamond = mean, white line = median, boxes = middle upper (top) and middle lower (bottom) quartile of the cell population, whiskers = highest (above) and lowest (below) migration speeds. Differences in HT-1080 morphology were determined by comparing quantified (**E**) circularity and (**F**) fraction of rounded cells (Elongation < 2.0). Circularity was calculated for individual cells (3 separate experiments, N > 200); Fraction of rounded cells was calculated per gel (at least two separate experiments, N ≥ 3 gels), with error bars representing standard deviation (*** = p < 0.001).

To determine the influence of 3D ECM properties on tumor cell motility (Figures 8-9, Figure S1), we compared quantified migration and morphologies for HT-1080s as a function of adhesion ligand density (250-1500 μM RGD), mechanical properties (220 Pa or 140 Pa), and culture platform (synthetic ECM compared to collagen). HT-1080 motility required RGD-mediated adhesion in synthetic ECM since cells were non-motile when RGD was replaced with non-bioactive RDG (Movie S14). HT-1080s migrated with weak RGD-dependence for cell speed (Figure 8A) and directionality (Figure 8B) in synthetic ECM, while results were similar in lower modulus synthetic ECM (140 Pa) compared the higher modulus (220 Pa) formulation (Figure S1). Finally, HT-1080 morphologies became more rounded (or less elongated) with decreasing RGD concentration or matrix modulus (Figure 8C). Strikingly, HT-1080s migrated in lower modulus synthetic ECM (Figure 9A; Movie S15) and collagen (Figure 9B; Movie S9) with statistically equivalent quantified speed (Figure 9C) and directionality (Figure 9D) despite an ~1.8-fold change in circularity (Figure 9E) and ~2.5-fold difference in the fraction of rounded cells (Figure 9F). Therefore, quantified HT-1080 motility was weakly influenced by changes in biochemical and biophysical properties of the ECM, even when morphologies were substantially altered. 

## Discussion

HT-1080 fibrosarcoma cells (HT-1080s) are a malignant cell type [63-67] commonly studied as a model for 3D tumor cell motility [5-11]. While fibrosarcoma tumors are mesenchymal in origin [68], we previously identified differences in motility compared to primary human dermal fibroblasts (hDFs) [10], which we hypothesized was due to disrupted function induced by the transformed phenotype [63-67,70]. To test our hypothesis, we used engineered 2D [71] and 3D [10,50] culture platforms to quantitatively compare migration, morphologies, and adhesiveness for HT-1080s and hDFs. HT-1080s differed substantially from hDFs for each of the qualitative and quantitative features investigated, including migration, morphologies, adhesiveness, expression of adhesion proteins, and cytoskeletal structure. These differences were not limited to synthetic ECM, as HT-1080s were also distinct from hDFs when cultured in collagen (Figure S2) and on tissue culture polystyrene or RGD-functionalized self-assembled monolayer arrays (Figure S8). Our results demonstrate that HT-1080s are characterized by a distinct phenotype compared to primary hDFs in 2D and 3D culture.

Transformation to a malignant phenotype induces profound changes in cytoskeletal structure that include pronounced rounding, loss of organized F-actin filaments, and diffuse myosin expression [17-22]. Several previous studies have demonstrated that fibroblasts retain organized cytoskeletal structure in 3D culture [93-98], and hDFs in synthetic ECM were similarly characterized by spread, multipolar morphologies and organized actomyosin filaments (Figure 1). In contrast, HT-1080s adopted relatively more rounded morphologies than hDFs in synthetic ECM, and were characterized by cortically organized F-actin and delocalized expression of myosin II (Figures 1, 4-5), while morphological differences were also evident in collagen (Figure S2). Our results were not limited to 3D culture, as rounding and cortical F-actin organization became pronounced for HT-1080s on RGD-SAMs, especially at lower RGD densities where hDFs retained spread morphologies and organized F-actin structure (Figure S8). Therefore, HT-1080s were characterized by rounded morphologies and cortically organized F-actin that contrasted with hDFs in 2D and 3D culture, suggesting that these changes in cytoskeletal structure were representative of an inherent phenotype. 

Transformation also induces reorganized vinculin-containing adhesions [22-26] and perturbs integrin function [27-30], while decreased adhesiveness is a defining property for malignant cells [26,30-36]. In synthetic ECM, hDFs expressed vinculin in condensed features on the tips of protrusions (Figure 2), which is consistent with 3D adhesions described for fibroblasts in naturally-derived materials [94,95,99]. In contrast, HT-1080s were characterized by delocalized vinculin expression and β1-integrin on the tips of leading protrusions in synthetic ECM (Figure 2, Figures S4-S5), which agrees with previous results in collagen [5,9]. While HT-1080s expressed β1-integrin on protrusions in synthetic ECM, migration was minimally dependent on adhesion (Figure 8, Figure S1) and cells remained highly motile after treatment with combinations of β1- and β3-integrin blocking antibodies (Figure S4). HT-1080s were also quantitatively less adhesive than several primary adherent human cell types, as they required a higher RGD density for attachment to RGD-SAMs than hDFs here (Figure 2), as well as human mesenchymal stem cells (hMSCs) and human umbilical vein endothelial cells (HUVECs) that we previously investigated on identical arrays [71]. Transformation by Rous sarcoma virus was previously reported to reduce adhesiveness on fibronectin-coated surfaces by altering β1-integrin affinity [29], and the weakly-adherent properties for HT-1080s suggests that they also present integrins in a low affinity or otherwise altered state [27-30]. Our combined results demonstrate that HT-1080s were characterized by a reorganized F-actin cytoskeleton and altered adhesion characteristics compared to primary human adherent cells, which is consistent with a mechanistic role for properties of the transformed phenotype in driving 3D migration. 

Normal and tumorigenic cell types migrate through non-proteolytic, contractility-dependent mechanisms that facilitate traction without requirements for strong matrix adhesion [5,100-108], and our results suggest that HT-1080s migrate in synthetic ECM through analogous proteolytic mechanisms. Expansion and contraction of large bleb-like bulging protrusions correlated with cell body movement for HT-1080s in synthetic ECM (Figure S6; Movie S6), which resembled amoeboid mechanisms for inserting pseudopodia into existing matrix gaps to generate traction (e.g., “chimneying”) [100-102,105-107]. However, HT-1080s must generate their own matrix gaps in synthetic ECM (Movie S1), and MT1-MMP expression on bulged leading protrusions (Figure 6) suggested a role for expansion of these bleb-like features in proteolysis (Movie S6). HT-1080s were also characterized by diffuse myosin II expression on the cell body and in discrete bulges along leading protrusions (Figure 5, Figure S6), while pronounced blebbing was observed throughout the cell (Figure 4, Figure S5; Movies S5, S7), which is consistent with a cortical contractility-driven mechanism for generating hydrostatic forces at the front and rear [101]. Finally, HT-1080s formed constriction rings (Figure 5; Movie S3) or less pronounced periodic membrane folds (Figure S6; Movie S6) that remained fixed relative to the matrix and propagated from the front to the rear in synthetic ECM (also collagen; Movie S9), which resembled cortical contraction waves [102-104] or cortical actin waves [109]. Taken together, our results demonstrate that cortical contractility played a diverse role for proteolytic HT-1080 motility in synthetic ECM, which may have broader implications for understanding how tumor cells navigate dense matrices.

Tumor cells form pronounced uropod-like structures that play a role in polarization for contractility-dependent migration in 3D culture [72-75]. Both rounded and elongated HT-1080s and WM239a cells formed similar uropod-like structures that were enriched with melanoma cell adhesion molecule (MCAM/cd146/MUC18), β1-integrin, and F-actin (Figure 7, Figure S7). MCAM has been implicated in several mechanisms critical to tumor cell motility and metastasis [110-113], and was identified as a component of a “Wnt-receptor-actin-myosin-polarity (WRAMP)” structure that was linked to polarization and protrusion retraction for WM239a cells [72,79]. The uropod-like structure for melanoma cells has also been correlated to invasion in vitro [73-75] and lung colonization in vivo [73], which is notable since MCAM functions as a cell-cell adhesion molecule for endothelial cells [114] and has been implicated in homing and extravasation [115,116]. Migrating GFP-MCAM WM239a cells were morphologically similar to rounded MDA-MB-231 breast carcinoma cells that were previously characterized by cortical F-actin flow towards a pronounced uropod-like structure [75,76] and A375 melanoma cells that expressed an “ezrin-rich uropod-like structure (ERULS) [74]. Interestingly, the proposed mechanism for cortical flow towards the uropod-like structure described for MDA-MB-231 cells [75,76] resembled mechanisms that have been proposed to facilitate cytokinesis [117-120]. MCAM was enriched on the contractile ring during cytokinesis for dividing GFP-MCAM WM239a cells, and remained pinned on the rear of each migrating daughter cell at the site of abscission (Figure S9; Movie S13). HT-1080s also expressed similar components on the rear end structure and contractile ring (Figures S7 and S9), suggesting that the uropod-like feature observed for tumor cells may be structurally related to the cytokinesis machinery and/or the midbody [72,121]. These combined results indicate that uropod-like structures play a diverse role for invasive tumor cells, and could provide an important mechanistic link between aggressive tumorigenic cell types with diverse origins, morphological features, and proteolytic requirements.

Several mechanistic similarities between migration and cell division have been described [119,120,122-130], and genetic signatures enriched with mitosis genes have been identified as predictors of metastasis and poor survival for several human cancers [131-133]. We identified a linear relationship between cell division and average distance migrated for HT-1080s in synthetic ECM (Figure S9E), which is consistent with a recently reported correlation between proliferation and migration distance for several malignant melanoma cell lines in 2D culture [130]. The mitotic kinases Cdc2 (Cdk1) [127] and Aurora-A [124] have been implicated in migration for aggressive tumorigenic cell types, and it was suggested that normal mitotic function for Cdc2 might be “hijacked” by tumor cells to facilitate invasion [128]. Cdc2 is a regulator of mitosis that plays a role in cytoskeletal reorganization [129,134,135], and has been shown to directly induce cell rounding when microinjected into fibroblasts [135]. Rounded HT-1080s migrating on RGD-SAMs express Cdc2 similarly to dividing cells, but not spread cells (Figure S10), which is consistent with the previously reported role for Cdc2 in migration for aggressive tumorigenic cell lines (including HT-1080s) [127]. Further, daughter HT-1080 cells retained a rounded morphology and a polarized phenotype after mitosis (Figure S9F; Movie S3), whereas hDFs extended multiple protrusions during cytokinesis, while daughter cells appeared to migrate more randomly (Figure S9G-H; Movie S16). More generally, mitosis induces several changes in cell function that mirror transformation (and several observations for HT-1080s here), including reorganization of the F-actin cytoskeleton [17,129,135,136], altered adhesiveness [32,137] and integrin function [27,28,30,138], and regulation of external response to growth factors [3,139]. While further investigation would be required to definitively identify a role for mitosis or cytokinesis signaling in tumor cell motility, we speculate that de-regulated proliferation may abnormally alter migration mechanisms for tumor cells with aggressive tumorigenic phenotypes.

Finally, membrane shedding has been implicated in tumor progression due to transfer of genetic material, long range cell signaling, suppression of the immune response, and transformation of fibroblasts and epithelial cells [140-144]. HT-1080s shed cell fragments of various sizes in collagen and synthetic ECM (Figures S4, S11; Movies S17, S18), including larger features that were several microns in diameter (e.g., Figure S4D-F). HT-1080s also formed secondary protrusions that split from the leading edge (Figure S11A-B; Movie S19) and remained attached to the cell body through a pronounced structure that resembled an intracellular bridge during cytokinesis (Figure S11D). The secondary protrusion for HT-1080s typically shifted around the cell body and was retracted at the rear (Figure S11A-B; Movie S19). However, protrusions often detached from the primary cell and migrated independently despite lacking a nucleus, and similar fragmenting was observed for HT-1080s in collagen (Figure S11E-H; Movies S17, S18). “Independent motile microplasts” (IMMPs) were previously identified for glioma cells in collagen, and it was reported that these features migrated for several days after being formed [144]. The frequency of IMMP formation was reported to increase as a function of invasiveness (based on a comparison of cell type) and ionizing radiation [144], while our results identified a correlation to RGD concentration (i.e., adhesion) for HT-1080s in synthetic ECM (Figure S11G). Therefore, while little is currently known about IMMPs, these large anucleate features facilitated all aspects of proteolytic motility for HT-1080s in synthetic ECM, and demonstrate a profoundly unstable phenotype that to the best of our knowledge has not been observed for normal primary cells. 

## Conclusion

Here, we compared migration and morphologies for HT-1080 fibrosarcoma cells and primary human dermal fibroblasts (hDFs) using engineered culture platforms to provide defined biochemical and biophysical matrix properties. In synthetic ECM, hDFs were characterized by several features that were consistent with those previously reported for fibroblasts in 3D culture [92-98], including multipolar morphologies, organized actomyosin filaments, and vinculin enrichment on the tips of protrusions. HT-1080s were characterized by cytoskeletal and adhesion properties that were distinct from hDFs in 2D and 3D culture, including quantitatively more rounded morphologies, decreased adhesiveness, and increased directional motility. Rounded and elongated HT-1080s also migrated through contractility-dependent, proteolytic mechanisms in synthetic ECM, and were characterized by cortical F-actin expression, cortex rupture and blebbing, cortical contraction waves or constriction rings, and a prominent rear end uropod-like structure. Our results demonstrate that HT-1080s adopt a distinct phenotype compared to hDFs under a wide range of 2D and 3D culture conditions.

Based on our results, we propose that aggressive tumorigenic cell types migrate distinctly from normal primary cells due to profound cellular changes induced by the transformed phenotype. HT-1080s were characterized by several features that were consistent with transformation, including a cortically organized F-actin cytoskeleton [17-22], diffuse vinculin expression [22-26], and a weakly-adherent phenotype [26,30-36]. Further, HT-1080s formed “independent motile microplasts (IMMPs)”, large migrating anucleate fragments that were previously correlated to increased invasiveness and exposure to ionizing radiation for glioma cells [144], pointing to a highly unstable tumorigenic phenotype that to the best of our knowledge has not been reported for primary cell types. Rounded and elongated HT-1080s and WM239a cells migrated through proteolytic modes and expressed prominent uropod-like structures that were similar to features reported for rounded A375 melanoma and MDA-MB-231 breast carcinoma cells migrating through non-proteolytic mechanisms [73-75]. Notably, HT-1080 fibrosarcoma cells, WM239a and A375 melanoma cells, and MDA-MB-231 breast carcinoma cells are each characterized by activating Raf or Ras mutations [63-66,145,146]. Ras and Raf regulate many cellular processes and are two of the most commonly mutated genes for metastatic cancers [1,3,147-150]. Importantly, the N-Ras mutation for HT-1080s disrupts cytoskeletal structure and activates several signaling pathways important for migration, including RhoA, Rac1, phosphoinositide 3-kinase (PI3K), and mitogen-activated protein kinase (MAPK) [63-66]. We speculate that activated signaling associated with mutated oncogenes (such as Ras or Raf) induces abnormal overlap between mechanisms that would otherwise be tightly regulated (such as migration and mitosis, see Discussion). Therefore, mutations common to metastatic cancers may provide a key starting point for elucidating migration mechanisms unique to aggressive tumorigenic cell types with diverse origins. 

## Supporting Information

Figure S1 **Quantified migration and morphologies for HT-1080 fibrosarcoma cells (HT-1080s) and human dermal fibroblasts (hDFs) in synthetic ECM.** (**A**) A comparison of directionality (DTO/TD, black) and persistence time (gray) as a function of RGD concentration for HT-1080s cultured in synthetic ECM (220 Pa). Distance-to-Origin (DTO) is the distance a cell moves from the initial starting position after 6 hours of tracking. Total distance (TD) is the sum of all individual movements during the 6 hours of tracking (15 min / frame, see Schematic). Persistence (*P*) was determined by fitting mean-squared displacement (MSD) data to a persistent random walk model (Dickinson, R.B. and R.T. Tranquillo, Aiche Journal 1993): .
MSD = 2S^2^P[t-P(1 - e^-t/P^)].
A sliding window algorithm was used to calculate mean squared displacements (MSD) at 15 minute time intervals (*t*), with speed (*S*) calculated as an unrestricted variable. All fits had R^2^ > 0.9 and numbers obtained were within a 95% confidence interval.
Quantified (**B**) cell speed and (**C**) DTO/TD as a function of RGD concentration for HT-1080s compared at two different matrix moduli (140 Pa and 220 Pa). (**D**) Average cell length as a function of RGD concentration for HT-1080s (black) and hDFs (gray). (**E**) Fraction of migrating HT-1080s (black) and hDFs (gray) as a function of RGD concentration (modulus = 220 Pa; all cells). All experiments: (≥ 6 gels, ≥ 40 cells migrating, at least two separate experiments).
(TIF)

Figure S2 **A comparison of morphologies for HT-1080s and hDFs in collagen.** HT-1080s (right) and hDFs (left) encapsulated in collagen (Type I, rat tail collagen, BD Biosciences) of varying densities (as labeled). (**A**-**D**) TRITC-conjugated phalloidin (F-actin, red) and DAPI (nucleus, blue). (**E**-**I**) Projected immunofluorescence images (Zeiss LSM Image Browser) illustrating β1-integrin (green), TRITC-conjugated phalloidin (F-actin, red) and DAPI (nucleus, blue). Insets in (E) and (F) illustrate HT-1080s from (I) and (G) on the same size scale as the hDFs shown.
(TIF)

Figure S3 **Analysis of punctate β1-integrin features for a human dermal fibroblast in synthetic ECM.** Immunofluorescence images illustrating a human dermal fibroblast (hDF): (**A**) Overlay; (**B**) F-actin (red, phalloidin); (**C**) β1-integrin (green). (**D**) Surface plot (Image J, “3D Surface Plot” plugin, See Methods) for β1-integrin expression to illustrate punctate features. (**E**) Illustration of the line profiles plotted in (F-K). (**F**-**K**) Profiles generated using the “Plot Profiles” command in Image J (grayscale pixel intensity on an 8-bit scale; 256 = saturation intensity). Features shown in (C) are also pointed out in (D, F-H). The relative grayscale was 2-5X higher for the punctate features (Lines 1-3) than maximum noise (Background 1-2).
(TIF)

Figure S4 **Influence of integrin blocking antibodies and illustration of β1-integrin dynamics at the rear for HT-1080s in synthetic ECM.** Normalized quantified (**A**) cell speed and (**B**) directionality (DTO/TD) for HT-1080s cultured in synthetic ECM (220 Pa, 1000 μM CRGDS). HT-1080s were treated with β1-integrin (Anti-b1) or β3-integrin (Anti-b3) blocking antibodies and a combination of both (Anti-b1,b3). β1-integrin blocking antibody (CD29, 6603113, Beckman Coulter) was added to the hydrogel monomer solution during polymerization (28 μg/mL final concentration) and to media (25 μg/mL final concentration) during swelling. For β3-integrin, 2 μL blocking antibody reagent (as received, CD-61, IM3605, Beckman Coulter) was added to monomer solution (PBS in monomer solution was decreased by same amount) and 30 μL was added to 1.5 mL media for overnight swelling. Media containing integrin blocking antibodies were exchanged with fresh solutions immediately prior to tracking migration. (**C**-**F**) Immunofluorescence images illustrating morphologies and rear end structures for HT-1080s cultured in synthetic ECM (220 Pa, 1000 μM CRGDS); β1-integrin (green), counterstained with TRITC-conjugated phalloidin (F-actin, red) and DAPI (nucleus, blue).
(TIF)

Figure S5 **Illustration of membrane folding and lateral blebbing for HT-1080s in synthetic ECM.** (**A**-**B**) Time-lapse images for an HT-1080 in synthetic ECM that is characterized by lateral blebbing (arrows) and pronounced wrinkling (See also Movie S7, time in Hr:Min:Sec). (**A**) 1 min/frame (00:13:30 to 00:25:30, every 4^th^ frame, from Movie S7). (**B**) 15 sec/frame (00:27:30 to 00:36:15, from Movie S7). Due to a change in procedure before revisions, synthetic ECM for the cell shown in (A,B) was prepared using 20,000 M.W. 8-arm poly(ethylene glycol)(PEG)-norbornene (40 mg/mL PEG-NB, 45% NB groups crosslinked with MMP-degradable peptide, 500 μM CRGDS) instead of 4-arm PEG-NB such as used for experiments shown in primary manuscript figures (Schematic, Figure 1A). Synthetic ECM was formed in roundbottom 96-well plates (7 μL / well) to image cells immediately after encapsulation while minimizing drift. Qualitatively similar results were obtained for morphological characterization using both hydrogel formulations. The HT-1080 in (A,B) is shown migrating in a region of the synthetic ECM that had not previously been degraded. Scale bars = 25 μm. (**C**-**E**) Immunofluorescence images for HT-1080s cultured in synthetic ECM (220 Pa, 1000 μM CRGDS); β1-integrin (green), counterstained with TRITC-conjugated phalloidin (F-actin, red) and DAPI (nucleus, blue). (**C**) Membrane ridges characterized by co-localized β1-integrin and F-actin were periodically expressed for HT-1080s in synthetic ECM. Overlay image (left panel) represents a projection of all planes (Zeiss Image Browser, maximum transparency) while images in the three panels to the right represent a single plane to better illustrate the radial features on the cell membrane. (**D**-**E**) HT-1080s in synthetic ECM were also characterized by membrane wrinkling (dashed boxes). (**E**) The top row represents a projection of all planes (Zeiss Image Browser, maximum transparency), while the bottom rows are two consecutive single plane images. ***Brightfield insets***: The HT-1080 in Movie S7 (also A and B) is compared to IF images during time frames that illustrate similar morphologies (time stamp from Movie S7 shown).
(TIF)

Figure S6 **Illustration of bleb-like bulges on leading protrusions for HT-1080s migrating in synthetic ECM.** Isotype control experiment comparing (**A**) rabbit myosin IIb antibody and (**B**) rabbit IgG control. Samples were treated using the procedure described in Methods (identical rabbit antibody concentration, myosin IIb or IgG control); Both samples were counterstained with Phalloidin (F-actin, red) and DAPI (not shown so that nuclear staining can be visualized) and then imaged using identical microscope settings. The IgG control antibody did not produce observable fluorescence, indicating that there was minimal expression due to non-specific effects. Therefore, myosin IIb expression within the nucleus and in the extracellular space is likely due to specific expression.
(**C**-**O**) Myosin II expression for HT-1080 cultured in synthetic ECM (220 Pa, 1000 μM CRGDS) (**C**) Z-projected immunofluorescence image illustrating myosin IIb (green), counterstained with TRITC-conjugated phalloidin (F-actin, red) and DAPI (nucleus, blue). Myosin II immunofluorescence expression for (**D**) Z-projection and (**E**-**I**) consecutive single planes. F-actin expression for (**J**) Z-projection and (**K**-**O**) consecutive single planes.
(**P**-**T**) Z-projected immunofluorescence image illustrating HT-1080s cultured in synthetic ECM (220 Pa, 1000 μM CRGDS): (**P**) MT1-MMP (MMP-14, green), counterstained with TRITC-conjugated phalloidin (F-actin, red) and DAPI (nucleus, blue). Single channel images for (**Q**) F-actin and (**R**) MT1-MMP. Images generated using ImageJ “Interactive 3D Surface Plot” function (“Fire” intensity scale) for (**S**) F-actin and (**T**) MT1-MMP.
(**U**) Expansion and contraction of bleb-like pseudopod on the front tip for an HT-1080 migrating in synthetic ECM (1 min / frame; 40 mg/mL 8-arm PEG-NB, 45% MMP crosslinks, 500 μM CRGDS; See also, Movie S6). Scale bar = 25 μm. ***Righttwopanels***: Comparison of (**V**) cell from time lapse images in (U) with (**W**) myosin IIb-expressing cell from (C).
(TIF)

Figure S7 **Uropod-like features and MCAM expression for tumor cells in synthetic ECM.** Z-projected immunofluorescence images (Zeiss LSM Image Browser, maximum transparency) for tumor cells in synthetic ECM (220 Pa, 1000 μM CRGDS unless otherwise noted). (**A**) WM239a melanoma cell: Melanoma cell adhesion molecule (MCAM/cd146/MUC18, green), counterstained with TRITC-conjugated phalloidin (F-actin, red) and DAPI (nucleus, blue). Image represents the rear of a cell that is oriented into the plane. Thin F-actin filaments appear to propagate radially from the uropod-like structure (white arrows). (**B**) WM239a melanoma cell: Myosin IIb (green), counterstained with TRITC-conjugated phalloidin (F-actin, red) and DAPI (nucleus, blue). Note that myosin IIb is expressed on the rear end uropod-like structure. (**C**,**D**) HT-1080 fibrosarcoma cells: Melanoma cell adhesion molecule (MCAM/cd146/MUC18, green), counterstained with TRITC-conjugated phalloidin (F-actin, red) and DAPI (nucleus, blue). HT-1080s shown in (C,D) were encapsulated in synthetic ECM formed with an 8-arm PEG-NB crosslinker (40,000 MW) with mechanical properties that were similar to the 4-arm PEG-NB used for most experiments. (**E**) HT-1080 fibrosarcoma cells: β1-integrin (green), counterstained with TRITC-conjugated phalloidin (F-actin, red) and DAPI (nucleus, blue). Cells shown in (D) and (E) illustrate similar morphological features and localization of MCAM and β1-integrin for apparent migrating daughter cells after cell division.
(TIF)

Figure S8 **A comparison of 2D morphologies for HT-1080s and hDFs.** (**A**,**B**) HT-1080s and (**C**,**D**) hDFs on RGD-SAMs (0.6% and 5% mol fraction RGD; Scale bar = 50 μm, A-D shown at the same magnification, except inset). ***Inset*** for (A) compares HT-1080s on RGD-SAM and in synthetic ECM (box = 30 x 30 μm). (**E**) Projected cell area for HT-1080s as a function of RGD density on RGD-SAMs. Error bars represent standard error of the mean for individual cells (* = p<0.05; ** = p<0.01). (**F**) HT-1080s and (**G**) hDFs on tissue culture polystyrene (TCP). (**H**) A comparison of migrating HT-1080s in synthetic ECM (3 wt% 20,000 MW 8-arm PEG-NB; 50% MMP crosslinks; 1 mM CRGDS) and on an RGD-SAM surface (1.7% RGD mol fraction). (**I**) hDFs on 0.02% mol fraction RGD-SAM spread and formed focal adhesions (HT-1080s did not attach at 0.02% mol fraction RGD). All immunofluorescence images illustrate vinculin (green), counterstained with phalloidin (F-actin, red) and DAPI (nucleus, blue).
(TIF)

Figure S9 **Polarity and cell division for cells in synthetic ECM.** (**A**) MCAM expression for a dividing GFP-MCAM WM239a melanoma cell. Upon completion of cell division, MCAM remains pinned on the rear of migrating daughter cells (See also, Movie S13). HT-1080s express myosin IIb on (**B**) the contractile ring and (**C**) the rear-end uropod like feature. Myosin IIb was expressed more clearly on the uropod-like feature for WM239a cells (e.g., Figure S7B). (**D**) Immunofluorescence images illustrating β1-integrin (green), counterstained with TRITC-conjugated phalloidin (F-actin, red) and DAPI (nucleus, blue) for a dividing HT-1080. (**E**) Average distance migrated (DTO, all cells) vs. cell division for HT-1080s in synthetic ECM (220 Pa, 250-1500 μM CRGDS). Average DTO was calculated for all cells (not just migrating cells), and provides a measure of effective invasiveness that accounts for speed and directionality. Successful cell division was defined as cells that rounded, formed a cleavage ring, and then separated into two distinct daughter cells. Migration and cell division were compared for the same 6 hour time course. Cells that began cell division before the 6 hours of tracking, or did not first undergo mitotic rounding, were not counted. There was a linear correlation between average DTO and successful cell division. Error bars represent standard error of the mean for individual hydrogels (≥ 8 gels, three separate experiments). (**F**) Time-lapse images (10 min / frame, Movie S3) illustrating a dividing HT-1080 in synthetic ECM (220 Pa, 1000 μM CRGDS). (**G**) Immunofluorescence images illustrating β1-integrin (green), counterstained with TRITC-conjugated phalloidin (F-actin, red) and DAPI (nucleus, blue) for a dividing hDF. (**H**) Time-lapse images (15 min / frame, also Movie S16) illustrating a dividing hDF in synthetic ECM (220 Pa, 1000 μM CRGDS). Notably, the hDF in (G) has begun to form substantial protrusions while the contractile ring is still prominent, which is consistent with time-lapse images in (H). Daughter HT-1080 cells (D) remain polarized and migrate persistently for several hours after division.
(TIF)

Figure S10 **Cdc2 expression for HT-1080s on RGD-SAMs.** (**A**) Time-lapse images (15 min / frame) illustrating HT-1080s on an RGD-SAM surface (1.7% mol fraction RGD). (**B**,**C**) Immunofluorescence images illustrating Cdc2 (Green), counterstained with TRITC-conjugated phalloidin (F-actin, red) and DAPI (nucleus, blue). Cells in (A) were fixed and are shown in (B). Images in (C) provide a broad overview for rounded and spread HT-1080s. Inset in (C) illustrates nuclei (DAPI) for cells during mitosis.
(TIF)

Figure S11 **HT-1080s adopt unstable phenotypes in 3D matrices.** (**A**,**B**) HT-1080s formed pronounced secondary protrusions (white arrows) that eventually retracted into the rear of the cell (See Movie S19). (**C**) Z-projected immunofluorescence images (Zeiss LSM Image Browser) for an HT-1080 in synthetic ECM (220 Pa, 1000 μM CRGDS) illustrating: Myosin IIb (green), counterstained with TRITC-conjugated phalloidin (F-actin, red) and DAPI (nucleus, blue). Rainbow intensity images are shown (to the right of false color images) to aid visualization of myosin IIb in the retracting feature at the rear of the cell. (**D**) Three separate z-projected immunofluorescence images (Zeiss LSM Image Browser) for HT-1080s in synthetic ECM (220 Pa, 1000 μM CRGDS) illustrating secondary features of various sizes; β1-integrin (green), counterstained with TRITC-conjugated phalloidin (F-actin, red) and DAPI (nucleus, blue). (**E**) Time-lapse images (1 hour / frame, inset = 15 min / frame; also Movie S17) illustrating formation of an “independent motile microplast” (IMMP, see Yount et. al, J. Neuro.-Oncol. 2007). Hydrogel was fixed and stained with DAPI (insets, final frame) to demonstrate that a nucleus was absent in the IMMP. (**F**) Immunofluorescence images illustrating an HT-1080 and an apparent IMMP in synthetic ECM (220 Pa, 1000 μM CRGDS); β1-integrin (green), counterstained with TRITC-conjugated phalloidin (F-actin, red) and DAPI (nucleus, blue). Image suggests that the IMMP migrated prior to fixing due to a trail of β1-integrin leading away from the HT-1080 containing a nucleus. Anucleate features of various sizes were common for HT-1080s, although motility cannot be definitively determined for IF images alone. (**G**) The fraction of HT-1080s characterized by abnormal fragmenting consistent with IMMP formation was proportional to RGD concentration (above 125 μM CRGDS). Cells were analyzed during the same 6 hour time period used for quantifying migration and cell division. Apparent IMMPs were determined based on cells that did not stop or exhibit rounding before splitting into two migrating features, as would be expected for normal cell division. While several IMMPs were confirmed by fixing and DAPI staining, most of the secondary features used for quantification were unconfirmed. (**H**) A migrating HT-1080 in collagen (15 min / frame) splits into two motile fragments without mitotic rounding (also Movie S18). IMMP formation was not confirmed in collagen, and therefore could be due to other abnormal cell division phenomena.
(TIF)

Movie S1 **Treatment with matrix metalloproteinase inhibitor (GM6001) blocks migration for HT-1080 fibrosarcoma cells in synthetic ECM.** Time-lapse images (15 min / frame) illustrating HT-1080s migrating in synthetic ECM (220 Pa, 1000 μM CRGDS) after treatment with DMSO (control, left) or matrix metalloproteinase (MMP) inhibitor (GM6001, right). Images represent minimum intensity z-projections (middle 500 μm of ~1000 μm thick matrix). HT-1080s are shown for the same time frame used for tracking and quantification of motility. No HT-1080s moved more than one cell length after treatment with MMP-inhibitor, demonstrating that proteolysis is required for migration in synthetic ECM. Time shown in Hr:Min. Scale bar = 100 μm.
(AVI)

Movie S2 **Human dermal fibroblast migrating in synthetic ECM.** Time-lapse images (10 min / frame) for a human dermal fibroblast (hDF) migrating in synthetic ECM (220 Pa, 1000 μM CRGDS). The hDF illustrated here is shown in Figure 3A (movie shown without image processing). Movement is consistent with hDF extension, attachment, cell body translocation, and rear end release (see Figure 3A). Time shown in Hr:Min. Scale bar = 25 μm.
(AVI)

Movie S3 **HT-1080 fibrosarcoma cell division and migration of daughter cells in synthetic ECM.** Time-lapse images (10 min / frame) illustrating cell division for an HT-1080 fibrosarcoma cell and migration of daughter cells in synthetic ECM (220 Pa, 1000 μM CRGDS). The same dividing HT-1080 is also illustrated in Figure S9F, and the daughter cell migrating towards the bottom, left is shown in Figures 3B (relationship between rear retraction and leading edge extension) and 5A (constriction ring formation). The cell migrating towards the top, right also illustrates formation of a split protrusion and retraction at the rear (beginning at 2:59, frame 19). Time shown in Hr:Min. Scale bar = 25 μm.
(AVI)

Movie S4 **Human dermal fibroblasts and HT-1080 fibrosarcoma cells migrating in synthetic ECM.** Time-lapse images (15 min / frame) illustrating human dermal fibroblasts (left) and HT-1080 fibrosarcoma cells (right) migrating in synthetic ECM (220 Pa, 1000 μM CRGDS). Images represent minimum intensity z-projections (200 μm stacks in the middle of ~ 1000 μm thick hydrogels). Time shown in Hr:Min. Scale bar = 100 μm.
(AVI)

Movie S5 **Blebbing on the leading protrusion for an HT-1080 fibrosarcoma cell migrating in synthetic ECM.** Time-lapse images (15 sec / frame) for an HT-1080 fibrosarcoma cell migrating in synthetic ECM (40 mg/mL 20,000 MW 8-arm PEG-NB, 50% MMP-degradable crosslinking density, 500 μM CRGDS). Time shown in Hr:Min:Sec. Scale bar = 25 μm.
(AVI)

Movie S6 **Dynamics of expanding and contracting bleb-like leading pseudopodia and corresponding movement of the cell body for an HT-1080 fibrosarcoma cell migrating in synthetic ECM.** Time-lapse images (60 sec / frame) for an HT-1080 fibrosarcoma cell migrating in synthetic ECM (40 mg/mL 20,000 MW 8-arm PEG-NB, 50% MMP-degradable crosslinking density, 500 μM CRGDS). Semi-spherical bleb-like bulges form at the leading edge as the cell body cortically contracts (e.g., 16:00-28:00). The rear of the cell moves forward as the bleb-like feature contracts (e.g., 29:00-36:00). The same HT-1080 is also illustrated in Figure S6U. Time shown in Min:Sec. Scale bar = 25 μm.
(AVI)

Movie S7 **Protrusion expansion, blebbing and cortex compression for an HT-1080 fibrosarcoma cell migrating in synthetic ECM.** Time-lapse images (15 sec / frame) for an HT-1080 in synthetic ECM (40 mg/mL 20,000 MW 8-arm PEG-NB, 45% crosslinking with MMP-degradable peptide, 500 μM CRGDS). The HT-1080 shown here is characterized by cell body and lateral blebbing, pronounced wrinkling, and expansion of the leading protrusion. The three frames (from the left) represent consecutive focal planes, while the last frame (far right) is a minimum intensity z-projection. The same HT-1080 is illustrated in Figure S5. Time shown in Hr:Min:Sec. Scale bar = 25 μm.
(AVI)

Movie S8 **HT-1080 fibrosarcoma cells migrating on RGD-functionalized self-assembled monolayer (RGD-SAM) surface.** Time-lapse images (30 sec / frame) illustrating HT-1080 fibrosarcoma cells migrating on an RGD-SAM surface (1.7% mol. fraction GRGDSP). Time shown in Hr:Min:Sec. Scale bar = 25 μm.
(AVI)

Movie S9 **HT-1080 fibrosarcoma cells migrating in 1.7 mg/mL collagen.** Time-lapse images (15 min / frame) for a single image plane illustrating HT-1080s migrating in 1.7 mg/mL collagen (BD Biosciences, High concentration rat tail collagen). The same HT-1080 is illustrated in Figure 5B (03:45-04:45). Time shown in Hr:Min. Scale bar = 50 μm.
(AVI)

Movie S10 **Treatment with myosin II inhibitor (Blebbistatin) inhibits migration for HT-1080 fibrosarcoma cells in synthetic ECM.** Time-lapse images (15 min / frame) illustrating HT-1080s migrating in synthetic ECM (220 Pa, 1000 μM CRGDS) after treatment with DMSO (control, left) or myosin II inhibitor (10 μM Blebbistatin, right). Images represent minimum intensity z-projections (middle 500 μm of ~1000 μm thick matrix). Time shown in Hr:Min. Scale bar = 50 μm.
(AVI)

Movie S11 **HT-1080 fibrosarcoma cell migrating in synthetic ECM with a rounded morphology (day 1).** Time-lapse images (15 min / frame) for a rounded HT-1080 fibrosarcoma cell migrating in synthetic ECM (220 Pa, 1000 μM CRGDS). The same HT-1080 is illustrated in Figure 6A. Time shown in Hr:Min. Scale bar = 50 μm.
(AVI)

Movie S12 **HT-1080 fibrosarcoma cell migrating in synthetic ECM with a rounded morphology (day 0).** Time-lapse images (10 min / frame) for a rounded HT-1080 fibrosarcoma cell migrating in synthetic ECM (220 Pa, 1000 μM CRGDS). The HT-1080 is shown during initial migration after encapsulation. The same HT-1080 is illustrated in Figures 4G and 6C. Time shown in Hr:Min. Scale bar = 25 μm.
(AVI)

Movie S13 **GFP-MCAM WM239a melanoma cell division and migration of daughter cells in synthetic ECM.** Time-lapse images (30 min / frame) illustrating cell division for GFP-MCAM transfected WM239a melanoma cell and migration of daughter cells in synthetic ECM (220 Pa, 1000 μM CRGDS); Brightfield (top) and GFP-MCAM (bottom). During cell division, MCAM became delocalized, and was then re-expressed on the cleavage ring during cytokinesis. MCAM remained pinned on the rear for each daughter cell at the point of cleavage, suggesting a possible relationship to the cytokinesis machinery or midbody. Time shown in Hr:Min. Scale bar = 20 μm.
(AVI)

Movie S14 **HT-1080 fibrosarcoma cells in synthetic ECM with non-bioactive RDG peptide (0 RGD, non-adhesive).** Time-lapse images (15 min / frame) illustrating HT-1080s immobilized in synthetic ECM with non-bioactive pendant peptide (220 Pa, 1500 μM C***RDG***S, 0 μM C**RGD**S). Images shown for a single plane in the middle of a ~1000 μm thick hydrogel. No HT-1080s moved more than one cell length in non-adhesive synthetic ECM (at least 6 hours of tracking). Time shown in Hr:Min. Scale bar = 100 μm.
(AVI)

Movie S15 **HT-1080 fibrosarcoma cells migrating in 140 Pa synthetic ECM.** Time-lapse images (15 min / frame) illustrating HT-1080 fibrosarcoma cells migrating in lower modulus synthetic ECM (140 Pa, 1500 μM CRGDS). Images represent minimum intensity z-projections (300 μm thickness in the middle of ~1000 μm thick matrix). Time shown in Hr:Min. Scale bar = 100 μm.
(AVI)

Movie S16 **Cell division for a human dermal fibroblast in synthetic ECM.** Time-lapse images (15 min / frame) illustrating cell division for a human dermal fibroblast (hDF) in synthetic ECM (220 Pa, 1000 μM CRGDS). The same hDF is illustrated in Figure S9H. The hDF begins to form protrusions before daughter cells complete abscission, while the separating cells migrate in an apparent random fashion that is distinct from polarized HT-1080s (Movie S3) or WM239a cells (Movie S10). Time shown in Hr:Min. Scale bar = 25 μm.
(AVI)

Movie S17 **HT-1080s form independent motile microplasts (IMMPs) in synthetic ECM.** Time-lapse images (15 min / frame) illustrating the formation of an “independent motile microplast” (IMMP, see Yount et. al., J. Neuro-Oncol. 2007) by a migrating HT-1080 in synthetic ECM (220 Pa, 1000 μMm CRGDS). Synthetic ECM was fixed immediately after last time point and stained with DAPI to demonstrate that one cell retained a nucleus while the other was an IMMP (See Figure S10E). Time shown in Hr:Min. Scale bar = 50 μm.
(AVI)

Movie S18 **HT-1080s split into multiple migrating cells or cell fragments in collagen.** Time-lapse images (15 min / frame) illustrating a migrating HT-1080 in collagen (1.7 mg/mL) that splits into two motile fragments without mitotic rounding (also Figure S10H). The formation of multiple motile fragments by HT-1080s during migration resembled IMMP formation (see Yount et. al., J. Neuro-Oncol. 2007) such as demonstrated in synthetic ECM (Movie S17). However, IMMP formation was not confirmed in collagen, and therefore the observations shown here could be due to other abnormal cell division phenomena. Time shown in Hr:Min. Scale bar = 50 μm.
(AVI)

Movie S19 **Abnormal protrusion dynamics for an HT-1080 in synthetic ECM.** Time-lapse images (10 min / frame) illustrating protrusion dynamics for an HT-1080 migrating in synthetic ECM (220 Pa, 1000 μM CRGDS). The same HT-1080 is illustrated in Figure S10B. The HT-1080 shown here forms protrusions that split into pronounced features resembling IMMPs (Movie S17), but retracted back into the migrating cell. Time shown in Hr:Min. Scale bar = 25 μm.
(AVI)
