# Supplementary figures and images for: A Quantitative Comparison of Human HT-1080 Fibrosarcoma Cells and Primary Human Dermal Fibroblasts Identifies a 3D Migration Mechanism with Properties Unique to the Transformed Phenotype
Source: PLoS One. 2013 Dec 3;8(12):e81689. doi: 10.1371/journal.pone.0081689 (PMC3857815; doi:10.1371/journal.pone.0081689)

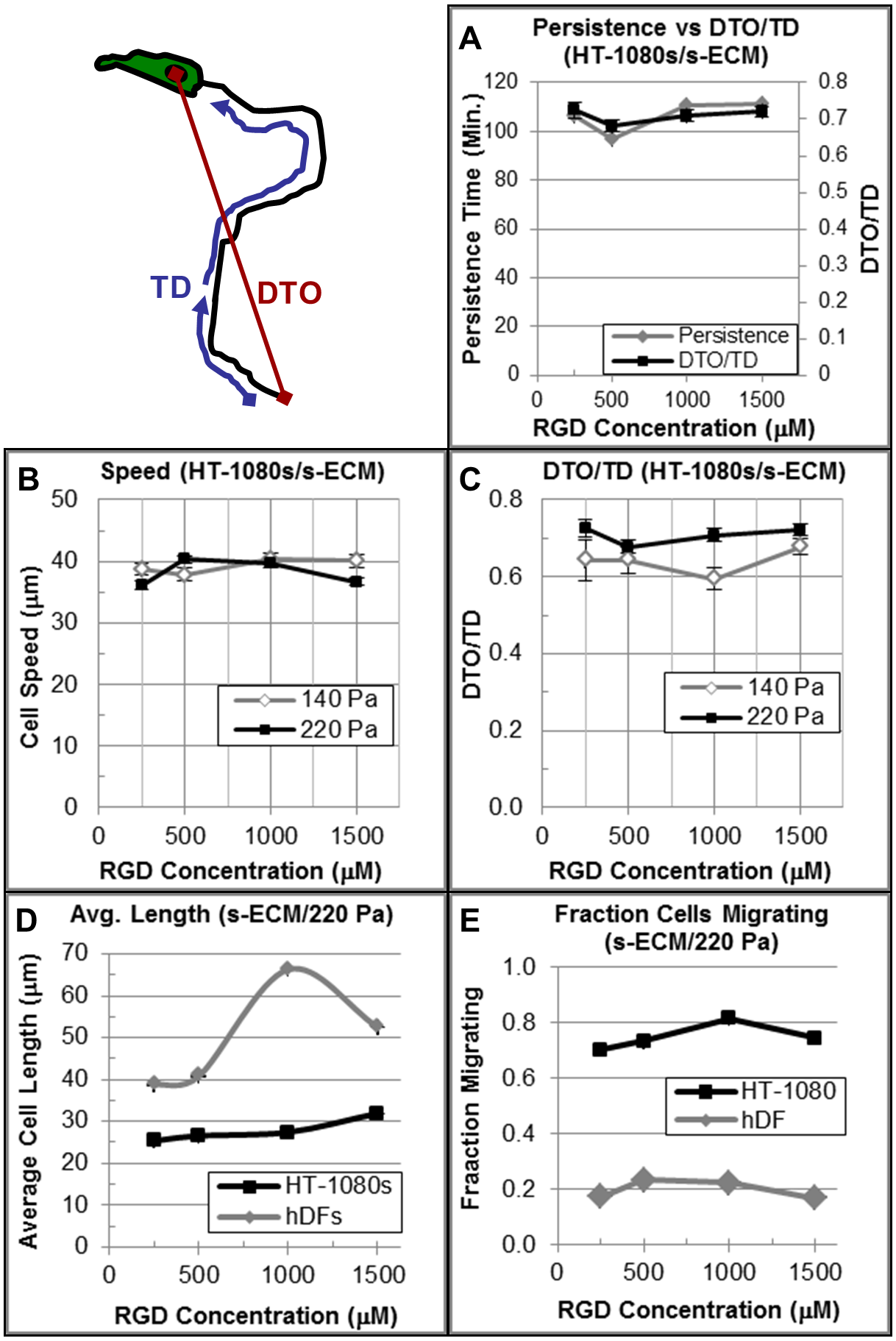

Supplement: Figure S1 — Quantified migration and morphologies for HT-1080 fibrosarcoma cells (HT-1080s) and human dermal fibroblasts (hDFs) in synthetic ECM. (A) A comparison of directionality (DTO/TD, black) and persistence time (gray) as a function of RGD concentration for HT-1080s cultured in synthetic ECM (220 Pa). Distance-to-Origin (DTO) is the distance a cell moves from the initial starting position after 6 hours of tracking. Total distance (TD) is the sum of all individual movements during the 6 hours of tracking (15 min / frame, see Schematic). Persistence (P) was determined by fitting mean-squared displacement (MSD) data to a persistent random walk model (Dickinson, R.B. and R.T. Tranquillo, Aiche Journal 1993): . MSD = 2S2P[t-P(1 - e-t/P)]. A sliding window algorithm was used to calculate mean squared displacements (MSD) at 15 minute time intervals (t), with speed (S) calculated as an unrestricted variable. All fits had R2 > 0.9 and numbers obtained were within a 95% confidence interval. Quantified (B) cell speed and (C) DTO/TD as a function of RGD concentration for HT-1080s compared at two different matrix moduli (140 Pa and 220 Pa). (D) Average cell length as a function of RGD concentration for HT-1080s (black) and hDFs (gray). (E) Fraction of migrating HT-1080s (black) and hDFs (gray) as a function of RGD concentration (modulus = 220 Pa; all cells). All experiments: (≥ 6 gels, ≥ 40 cells migrating, at least two separate experiments). (TIF) [file pone.0081689.s001.tif]

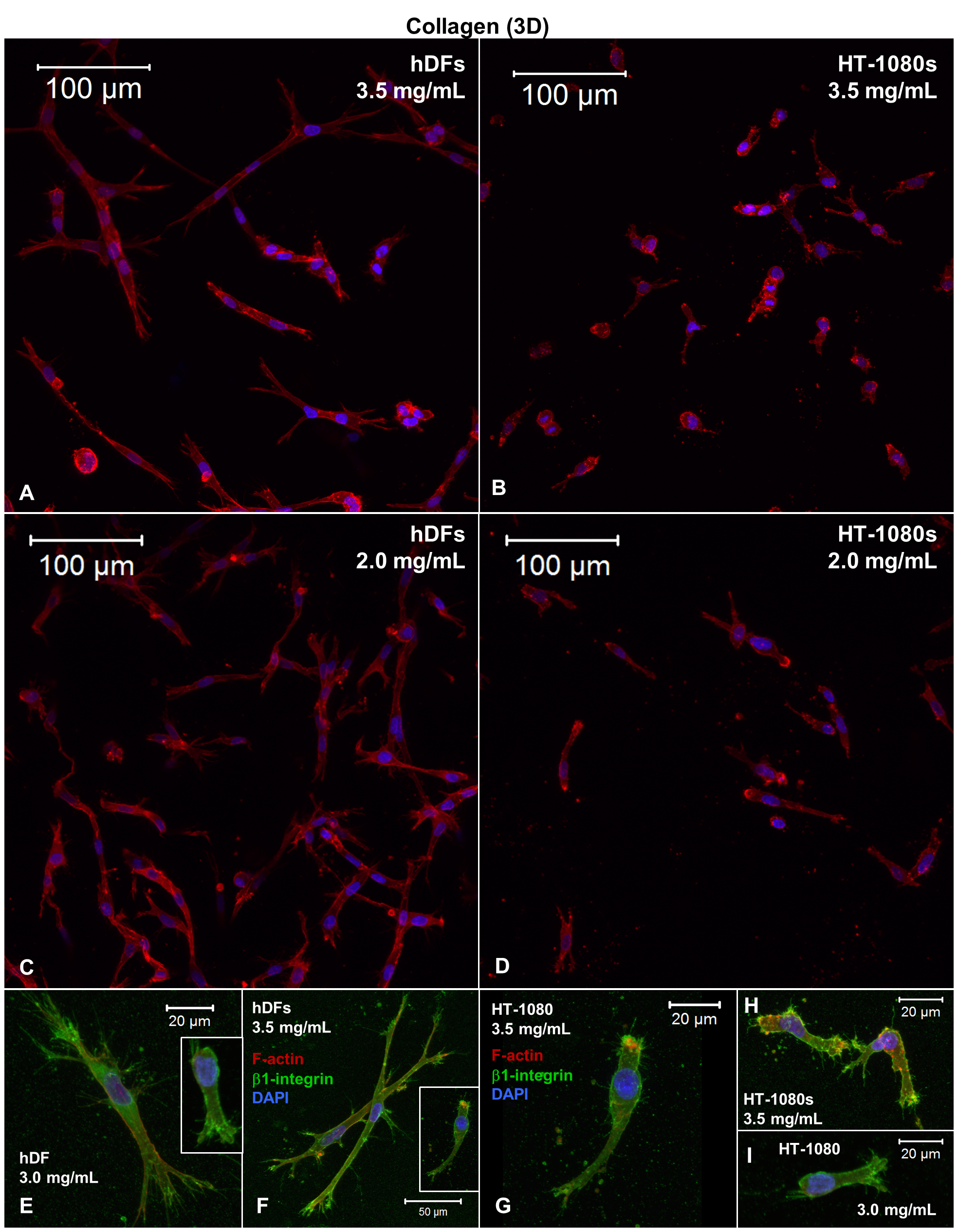

Supplement: Figure S2 — A comparison of morphologies for HT-1080s and hDFs in collagen. HT-1080s (right) and hDFs (left) encapsulated in collagen (Type I, rat tail collagen, BD Biosciences) of varying densities (as labeled). (A-D) TRITC-conjugated phalloidin (F-actin, red) and DAPI (nucleus, blue). (E-I) Projected immunofluorescence images (Zeiss LSM Image Browser) illustrating β1-integrin (green), TRITC-conjugated phalloidin (F-actin, red) and DAPI (nucleus, blue). Insets in (E) and (F) illustrate HT-1080s from (I) and (G) on the same size scale as the hDFs shown. (TIF) [file pone.0081689.s002.tif]

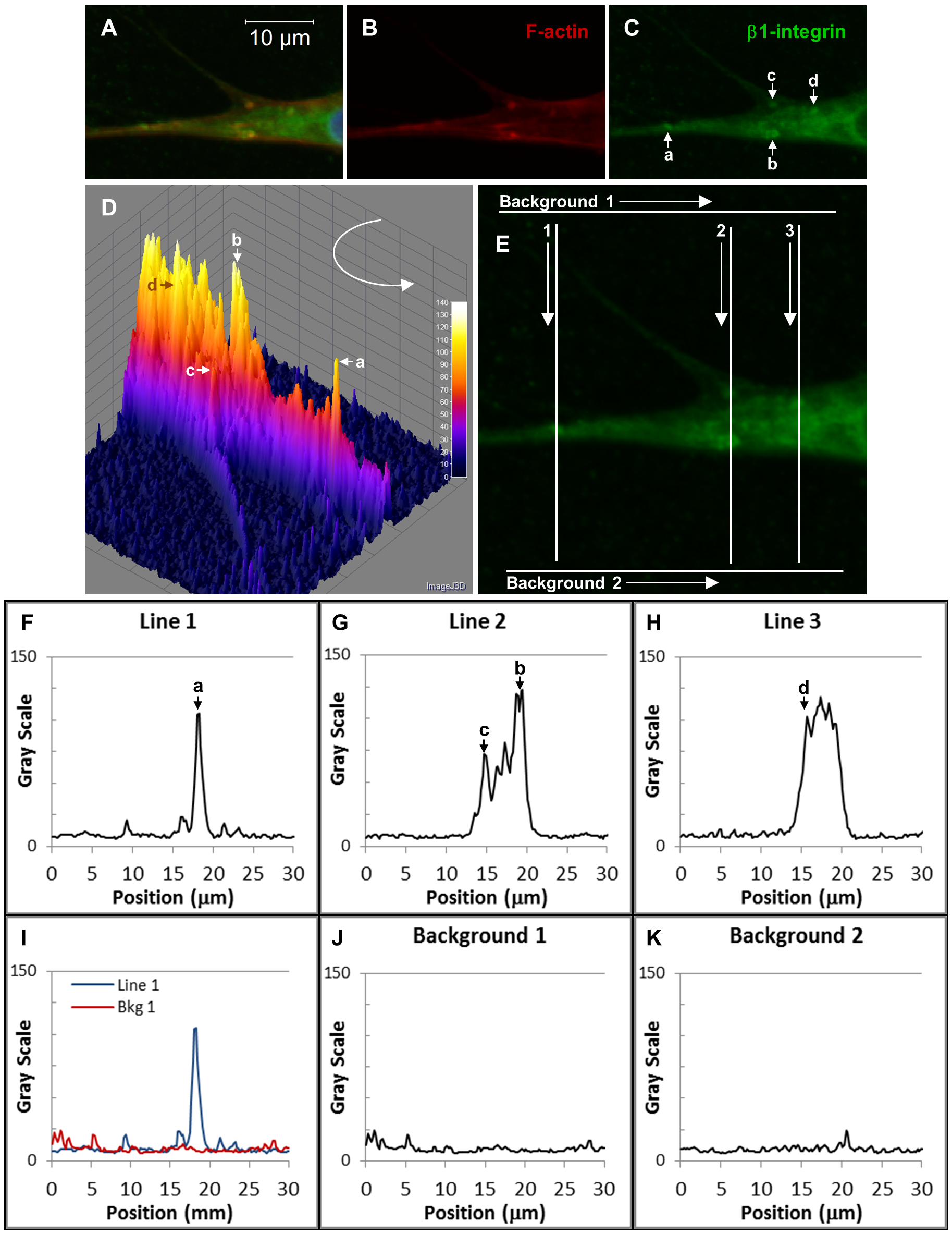

Supplement: Figure S3 — Analysis of punctate β1-integrin features for a human dermal fibroblast in synthetic ECM. Immunofluorescence images illustrating a human dermal fibroblast (hDF): (A) Overlay; (B) F-actin (red, phalloidin); (C) β1-integrin (green). (D) Surface plot (Image J, “3D Surface Plot” plugin, See Methods) for β1-integrin expression to illustrate punctate features. (E) Illustration of the line profiles plotted in (F-K). (F-K) Profiles generated using the “Plot Profiles” command in Image J (grayscale pixel intensity on an 8-bit scale; 256 = saturation intensity). Features shown in (C) are also pointed out in (D, F-H). The relative grayscale was 2-5X higher for the punctate features (Lines 1-3) than maximum noise (Background 1-2). (TIF) [file pone.0081689.s003.tif]

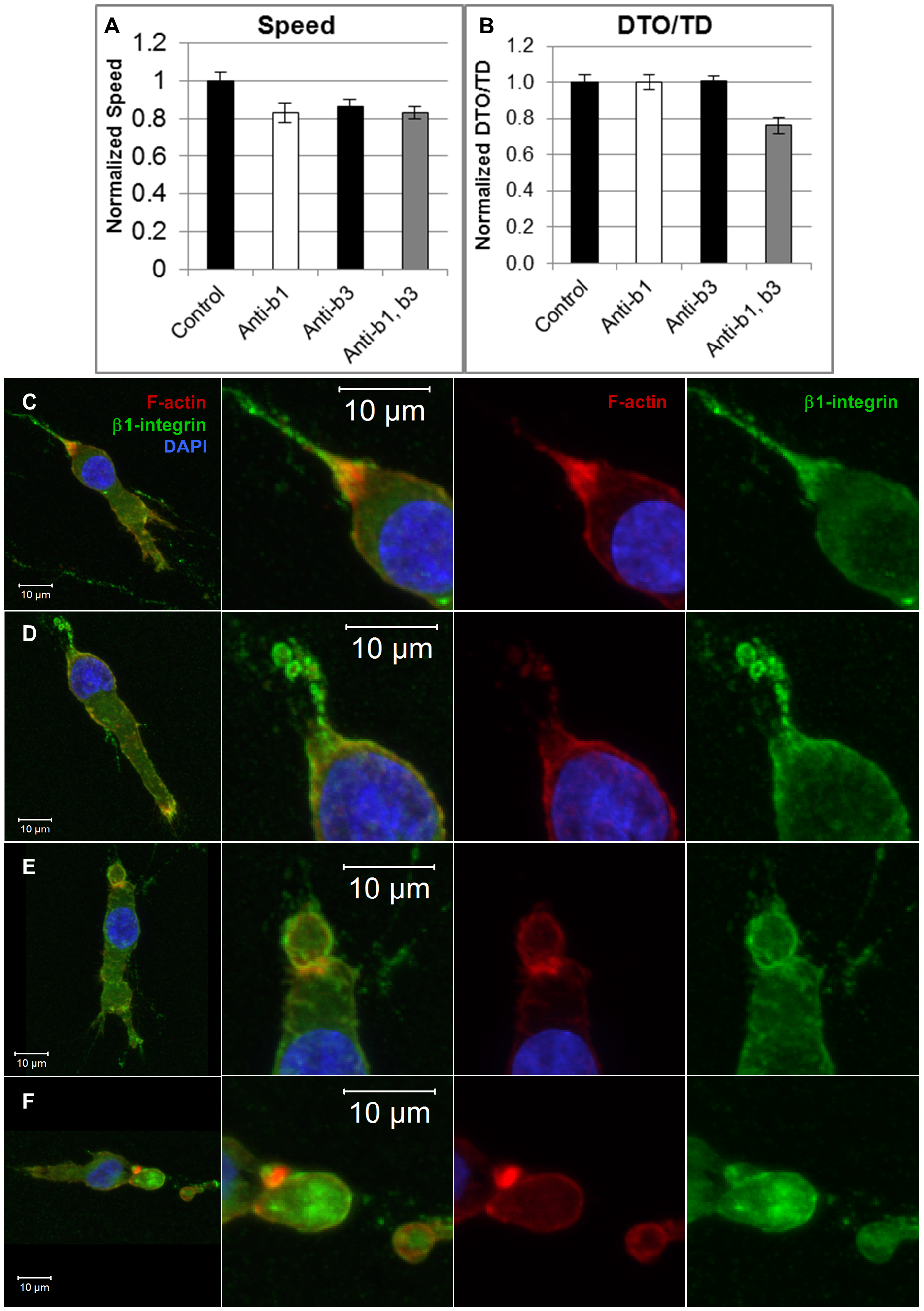

Supplement: Figure S4 — Influence of integrin blocking antibodies and illustration of β1-integrin dynamics at the rear for HT-1080s in synthetic ECM. Normalized quantified (A) cell speed and (B) directionality (DTO/TD) for HT-1080s cultured in synthetic ECM (220 Pa, 1000 μM CRGDS). HT-1080s were treated with β1-integrin (Anti-b1) or β3-integrin (Anti-b3) blocking antibodies and a combination of both (Anti-b1,b3). β1-integrin blocking antibody (CD29, 6603113, Beckman Coulter) was added to the hydrogel monomer solution during polymerization (28 μg/mL final concentration) and to media (25 μg/mL final concentration) during swelling. For β3-integrin, 2 μL blocking antibody reagent (as received, CD-61, IM3605, Beckman Coulter) was added to monomer solution (PBS in monomer solution was decreased by same amount) and 30 μL was added to 1.5 mL media for overnight swelling. Media containing integrin blocking antibodies were exchanged with fresh solutions immediately prior to tracking migration. (C-F) Immunofluorescence images illustrating morphologies and rear end structures for HT-1080s cultured in synthetic ECM (220 Pa, 1000 μM CRGDS); β1-integrin (green), counterstained with TRITC-conjugated phalloidin (F-actin, red) and DAPI (nucleus, blue). (TIF) [file pone.0081689.s004.tif]

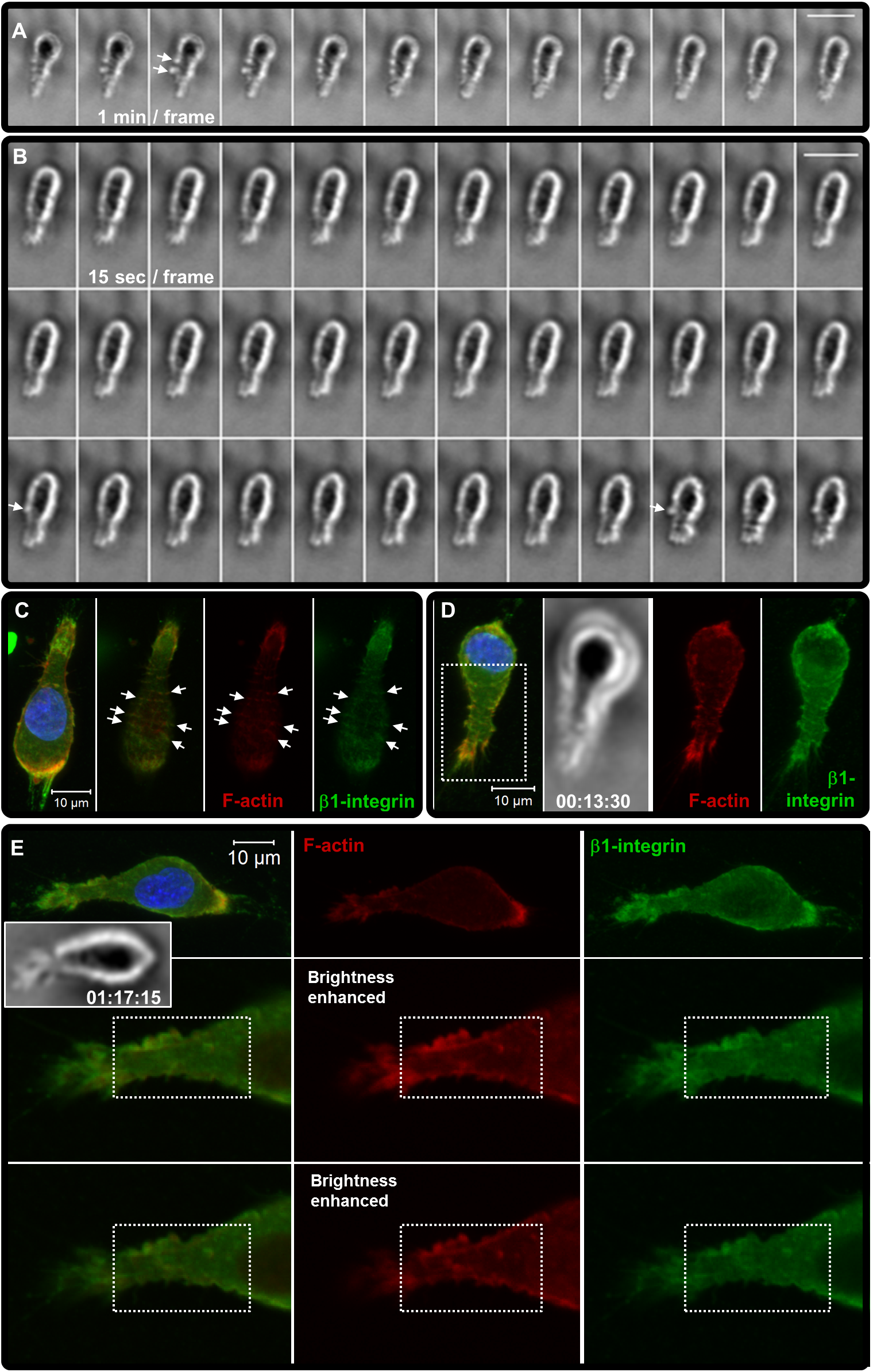

Supplement: Figure S5 — Illustration of membrane folding and lateral blebbing for HT-1080s in synthetic ECM. (A-B) Time-lapse images for an HT-1080 in synthetic ECM that is characterized by lateral blebbing (arrows) and pronounced wrinkling (See also Movie S7, time in Hr:Min:Sec). (A) 1 min/frame (00:13:30 to 00:25:30, every 4th frame, from Movie S7). (B) 15 sec/frame (00:27:30 to 00:36:15, from Movie S7). Due to a change in procedure before revisions, synthetic ECM for the cell shown in (A,B) was prepared using 20,000 M.W. 8-arm poly(ethylene glycol)(PEG)-norbornene (40 mg/mL PEG-NB, 45% NB groups crosslinked with MMP-degradable peptide, 500 μM CRGDS) instead of 4-arm PEG-NB such as used for experiments shown in primary manuscript figures (Schematic, Figure 1A). Synthetic ECM was formed in roundbottom 96-well plates (7 μL / well) to image cells immediately after encapsulation while minimizing drift. Qualitatively similar results were obtained for morphological characterization using both hydrogel formulations. The HT-1080 in (A,B) is shown migrating in a region of the synthetic ECM that had not previously been degraded. Scale bars = 25 μm. (C-E) Immunofluorescence images for HT-1080s cultured in synthetic ECM (220 Pa, 1000 μM CRGDS); β1-integrin (green), counterstained with TRITC-conjugated phalloidin (F-actin, red) and DAPI (nucleus, blue). (C) Membrane ridges characterized by co-localized β1-integrin and F-actin were periodically expressed for HT-1080s in synthetic ECM. Overlay image (left panel) represents a projection of all planes (Zeiss Image Browser, maximum transparency) while images in the three panels to the right represent a single plane to better illustrate the radial features on the cell membrane. (D-E) HT-1080s in synthetic ECM were also characterized by membrane wrinkling (dashed boxes). (E) The top row represents a projection of all planes (Zeiss Image Browser, maximum transparency), while the bottom rows are two consecutive single plane images. Brightfield insets: The HT- [file pone.0081689.s005.tif]

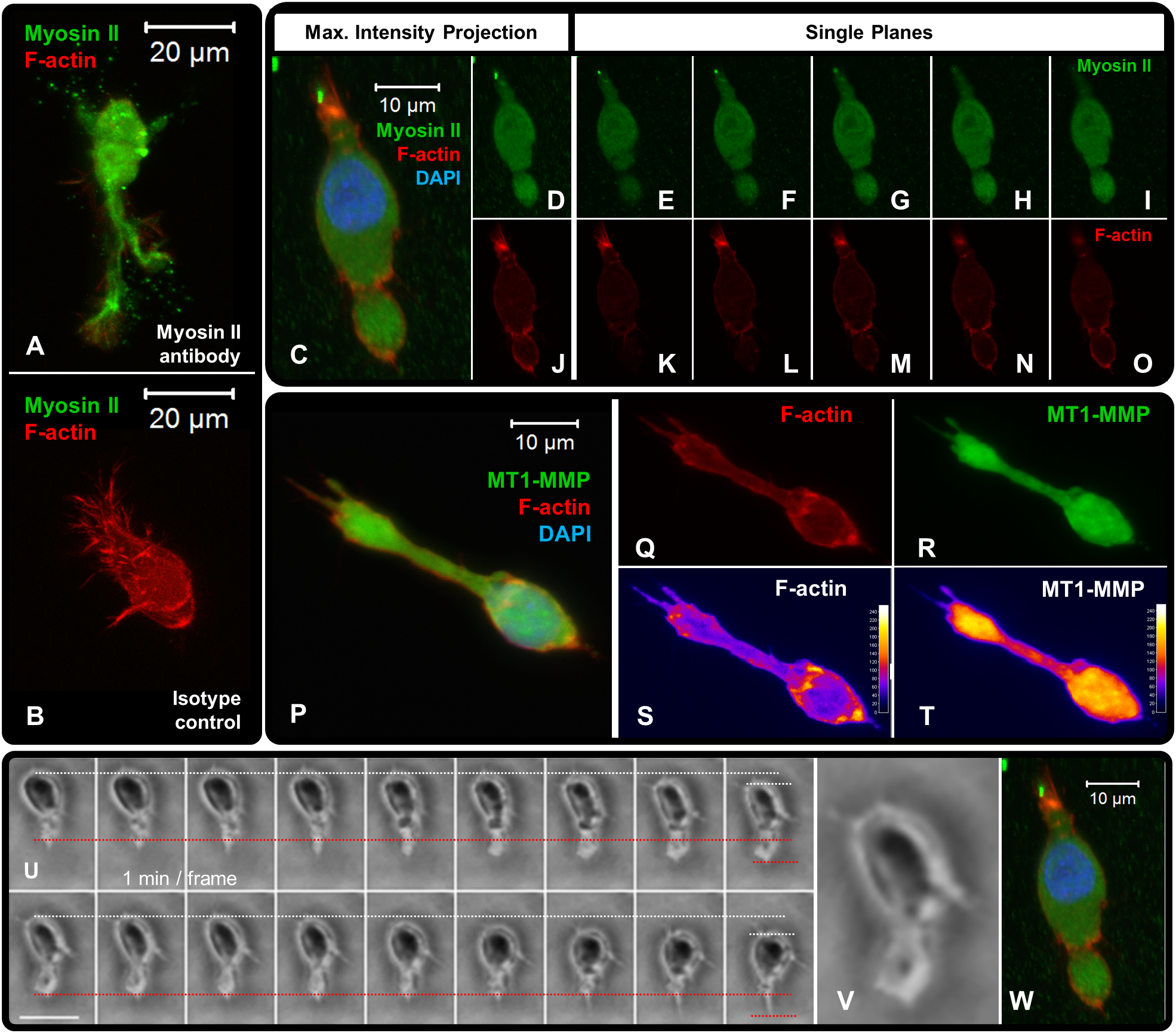

Supplement: Figure S6 — Illustration of bleb-like bulges on leading protrusions for HT-1080s migrating in synthetic ECM. Isotype control experiment comparing (A) rabbit myosin IIb antibody and (B) rabbit IgG control. Samples were treated using the procedure described in Methods (identical rabbit antibody concentration, myosin IIb or IgG control); Both samples were counterstained with Phalloidin (F-actin, red) and DAPI (not shown so that nuclear staining can be visualized) and then imaged using identical microscope settings. The IgG control antibody did not produce observable fluorescence, indicating that there was minimal expression due to non-specific effects. Therefore, myosin IIb expression within the nucleus and in the extracellular space is likely due to specific expression. (C-O) Myosin II expression for HT-1080 cultured in synthetic ECM (220 Pa, 1000 μM CRGDS) (C) Z-projected immunofluorescence image illustrating myosin IIb (green), counterstained with TRITC-conjugated phalloidin (F-actin, red) and DAPI (nucleus, blue). Myosin II immunofluorescence expression for (D) Z-projection and (E-I) consecutive single planes. F-actin expression for (J) Z-projection and (K-O) consecutive single planes. (P-T) Z-projected immunofluorescence image illustrating HT-1080s cultured in synthetic ECM (220 Pa, 1000 μM CRGDS): (P) MT1-MMP (MMP-14, green), counterstained with TRITC-conjugated phalloidin (F-actin, red) and DAPI (nucleus, blue). Single channel images for (Q) F-actin and (R) MT1-MMP. Images generated using ImageJ “Interactive 3D Surface Plot” function (“Fire” intensity scale) for (S) F-actin and (T) MT1-MMP. (U) Expansion and contraction of bleb-like pseudopod on the front tip for an HT-1080 migrating in synthetic ECM (1 min / frame; 40 mg/mL 8-arm PEG-NB, 45% MMP crosslinks, 500 μM CRGDS; See also, Movie S6). Scale bar = 25 μm. Righttwopanels: Comparison of (V) cell from time lapse images in (U) with (W) myosin IIb-expressing cell from (C). (TIF) [file pone.0081689.s006.tif]

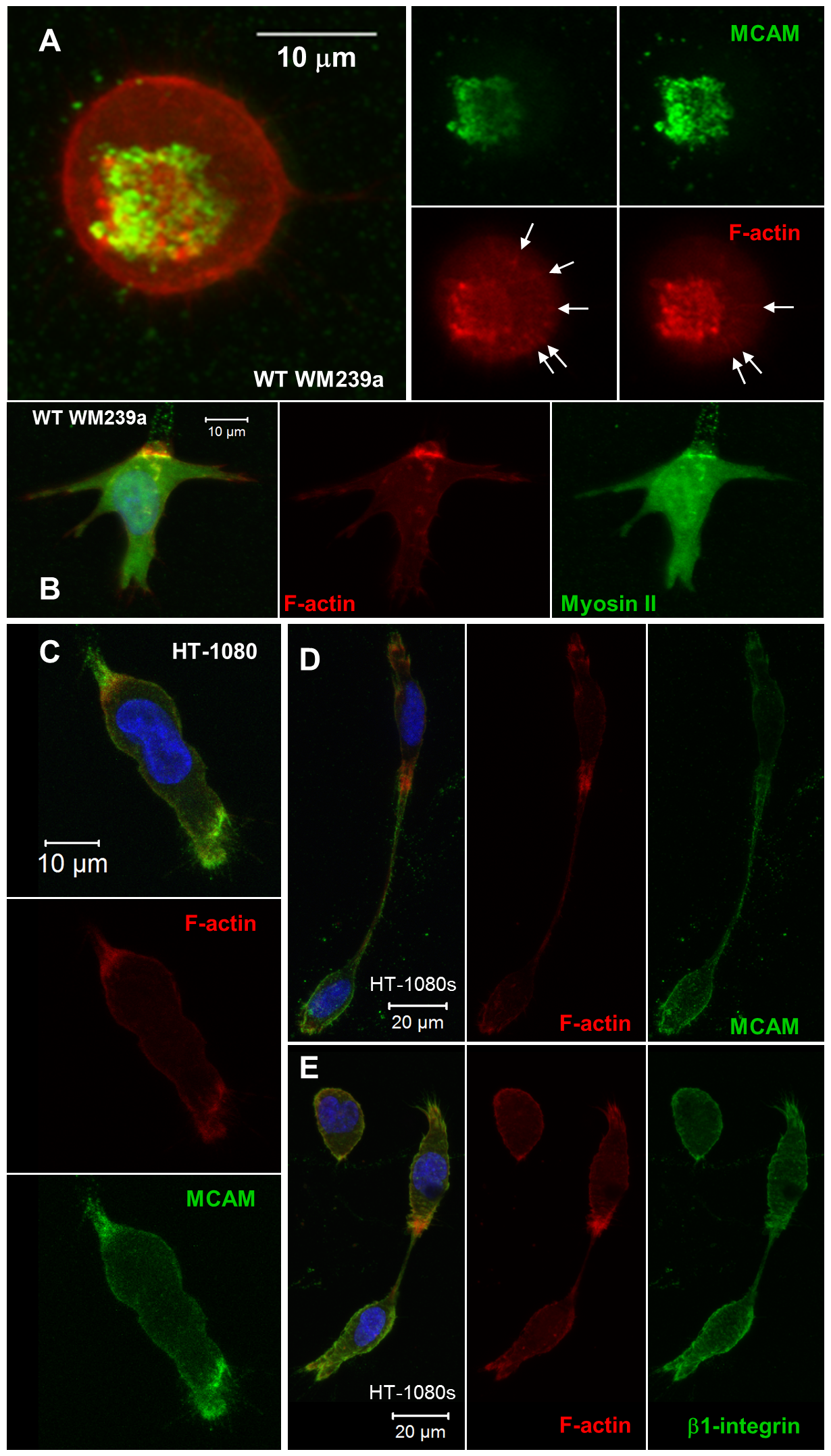

Supplement: Figure S7 — Uropod-like features and MCAM expression for tumor cells in synthetic ECM. Z-projected immunofluorescence images (Zeiss LSM Image Browser, maximum transparency) for tumor cells in synthetic ECM (220 Pa, 1000 μM CRGDS unless otherwise noted). (A) WM239a melanoma cell: Melanoma cell adhesion molecule (MCAM/cd146/MUC18, green), counterstained with TRITC-conjugated phalloidin (F-actin, red) and DAPI (nucleus, blue). Image represents the rear of a cell that is oriented into the plane. Thin F-actin filaments appear to propagate radially from the uropod-like structure (white arrows). (B) WM239a melanoma cell: Myosin IIb (green), counterstained with TRITC-conjugated phalloidin (F-actin, red) and DAPI (nucleus, blue). Note that myosin IIb is expressed on the rear end uropod-like structure. (C,D) HT-1080 fibrosarcoma cells: Melanoma cell adhesion molecule (MCAM/cd146/MUC18, green), counterstained with TRITC-conjugated phalloidin (F-actin, red) and DAPI (nucleus, blue). HT-1080s shown in (C,D) were encapsulated in synthetic ECM formed with an 8-arm PEG-NB crosslinker (40,000 MW) with mechanical properties that were similar to the 4-arm PEG-NB used for most experiments. (E) HT-1080 fibrosarcoma cells: β1-integrin (green), counterstained with TRITC-conjugated phalloidin (F-actin, red) and DAPI (nucleus, blue). Cells shown in (D) and (E) illustrate similar morphological features and localization of MCAM and β1-integrin for apparent migrating daughter cells after cell division. (TIF) [file pone.0081689.s007.tif]

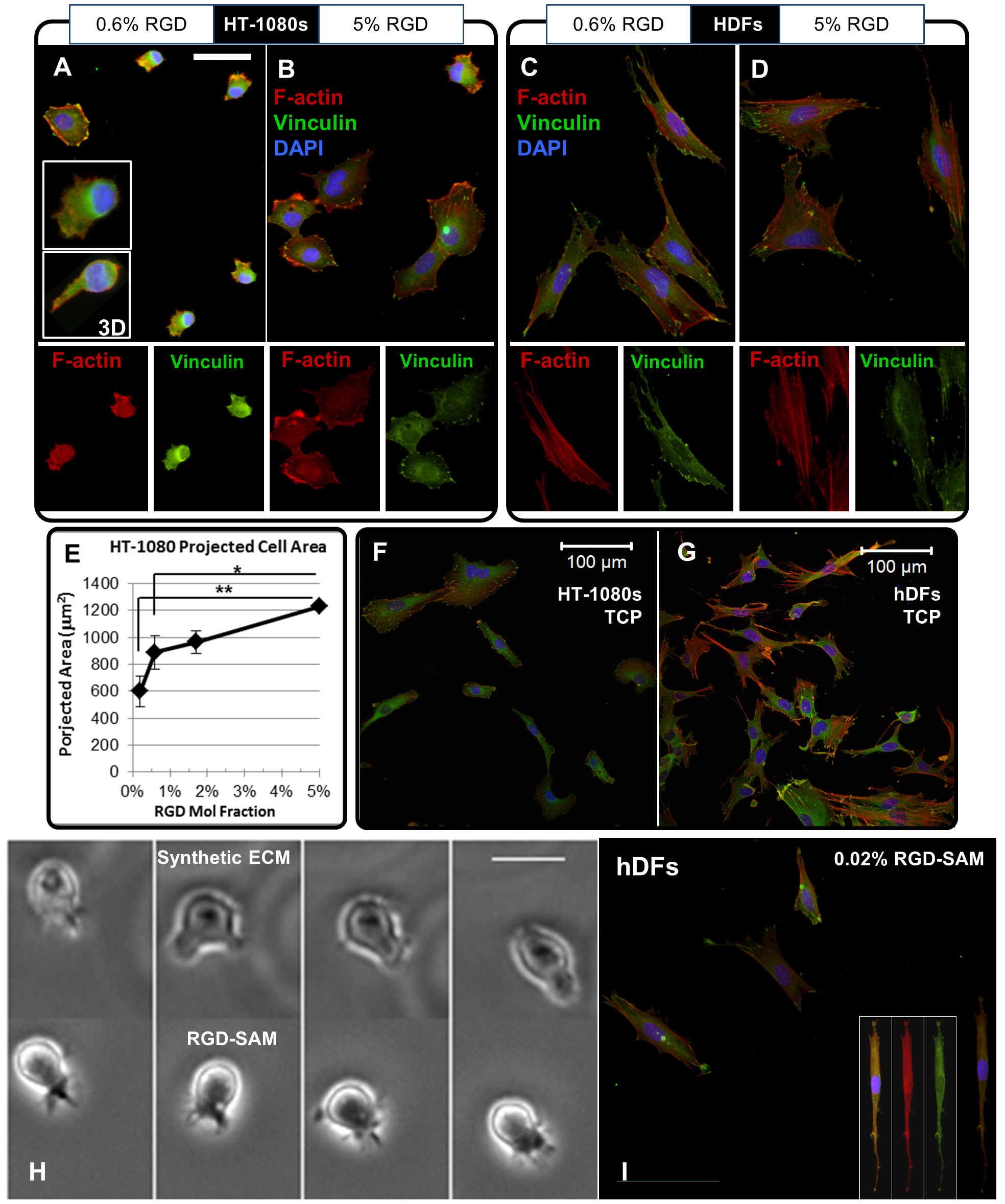

Supplement: Figure S8 — A comparison of 2D morphologies for HT-1080s and hDFs. (A,B) HT-1080s and (C,D) hDFs on RGD-SAMs (0.6% and 5% mol fraction RGD; Scale bar = 50 μm, A-D shown at the same magnification, except inset). Inset for (A) compares HT-1080s on RGD-SAM and in synthetic ECM (box = 30 x 30 μm). (E) Projected cell area for HT-1080s as a function of RGD density on RGD-SAMs. Error bars represent standard error of the mean for individual cells (* = p<0.05; ** = p<0.01). (F) HT-1080s and (G) hDFs on tissue culture polystyrene (TCP). (H) A comparison of migrating HT-1080s in synthetic ECM (3 wt% 20,000 MW 8-arm PEG-NB; 50% MMP crosslinks; 1 mM CRGDS) and on an RGD-SAM surface (1.7% RGD mol fraction). (I) hDFs on 0.02% mol fraction RGD-SAM spread and formed focal adhesions (HT-1080s did not attach at 0.02% mol fraction RGD). All immunofluorescence images illustrate vinculin (green), counterstained with phalloidin (F-actin, red) and DAPI (nucleus, blue). (TIF) [file pone.0081689.s008.tif]

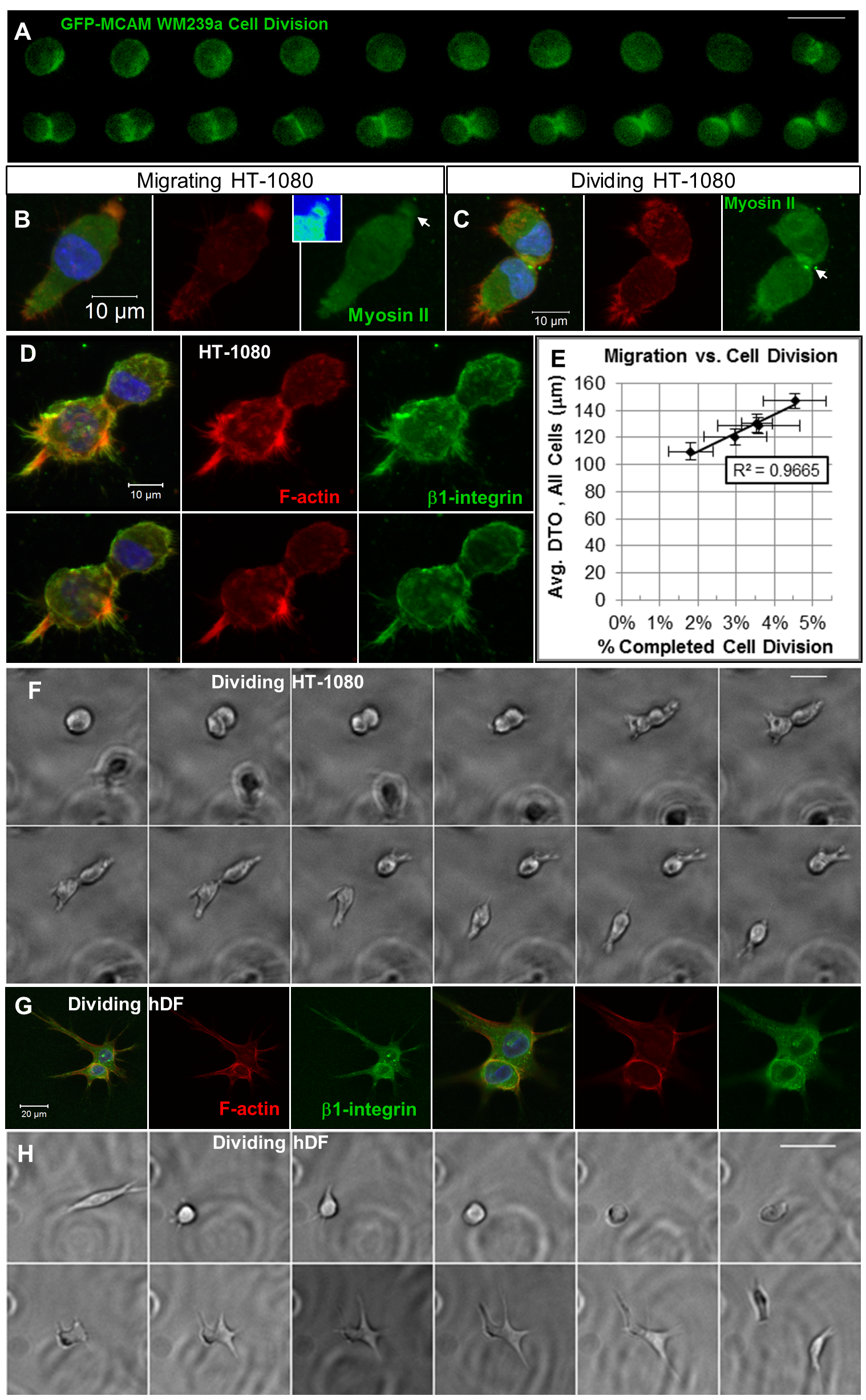

Supplement: Figure S9 — Polarity and cell division for cells in synthetic ECM. (A) MCAM expression for a dividing GFP-MCAM WM239a melanoma cell. Upon completion of cell division, MCAM remains pinned on the rear of migrating daughter cells (See also, Movie S13). HT-1080s express myosin IIb on (B) the contractile ring and (C) the rear-end uropod like feature. Myosin IIb was expressed more clearly on the uropod-like feature for WM239a cells (e.g., Figure S7B). (D) Immunofluorescence images illustrating β1-integrin (green), counterstained with TRITC-conjugated phalloidin (F-actin, red) and DAPI (nucleus, blue) for a dividing HT-1080. (E) Average distance migrated (DTO, all cells) vs. cell division for HT-1080s in synthetic ECM (220 Pa, 250-1500 μM CRGDS). Average DTO was calculated for all cells (not just migrating cells), and provides a measure of effective invasiveness that accounts for speed and directionality. Successful cell division was defined as cells that rounded, formed a cleavage ring, and then separated into two distinct daughter cells. Migration and cell division were compared for the same 6 hour time course. Cells that began cell division before the 6 hours of tracking, or did not first undergo mitotic rounding, were not counted. There was a linear correlation between average DTO and successful cell division. Error bars represent standard error of the mean for individual hydrogels (≥ 8 gels, three separate experiments). (F) Time-lapse images (10 min / frame, Movie S3) illustrating a dividing HT-1080 in synthetic ECM (220 Pa, 1000 μM CRGDS). (G) Immunofluorescence images illustrating β1-integrin (green), counterstained with TRITC-conjugated phalloidin (F-actin, red) and DAPI (nucleus, blue) for a dividing hDF. (H) Time-lapse images (15 min / frame, also Movie S16) illustrating a dividing hDF in synthetic ECM (220 Pa, 1000 μM CRGDS). Notably, the hDF in (G) has begun to form substantial protrusions while the contractile ring is still prominent, which is consistent with time-lapse i [file pone.0081689.s009.tif]

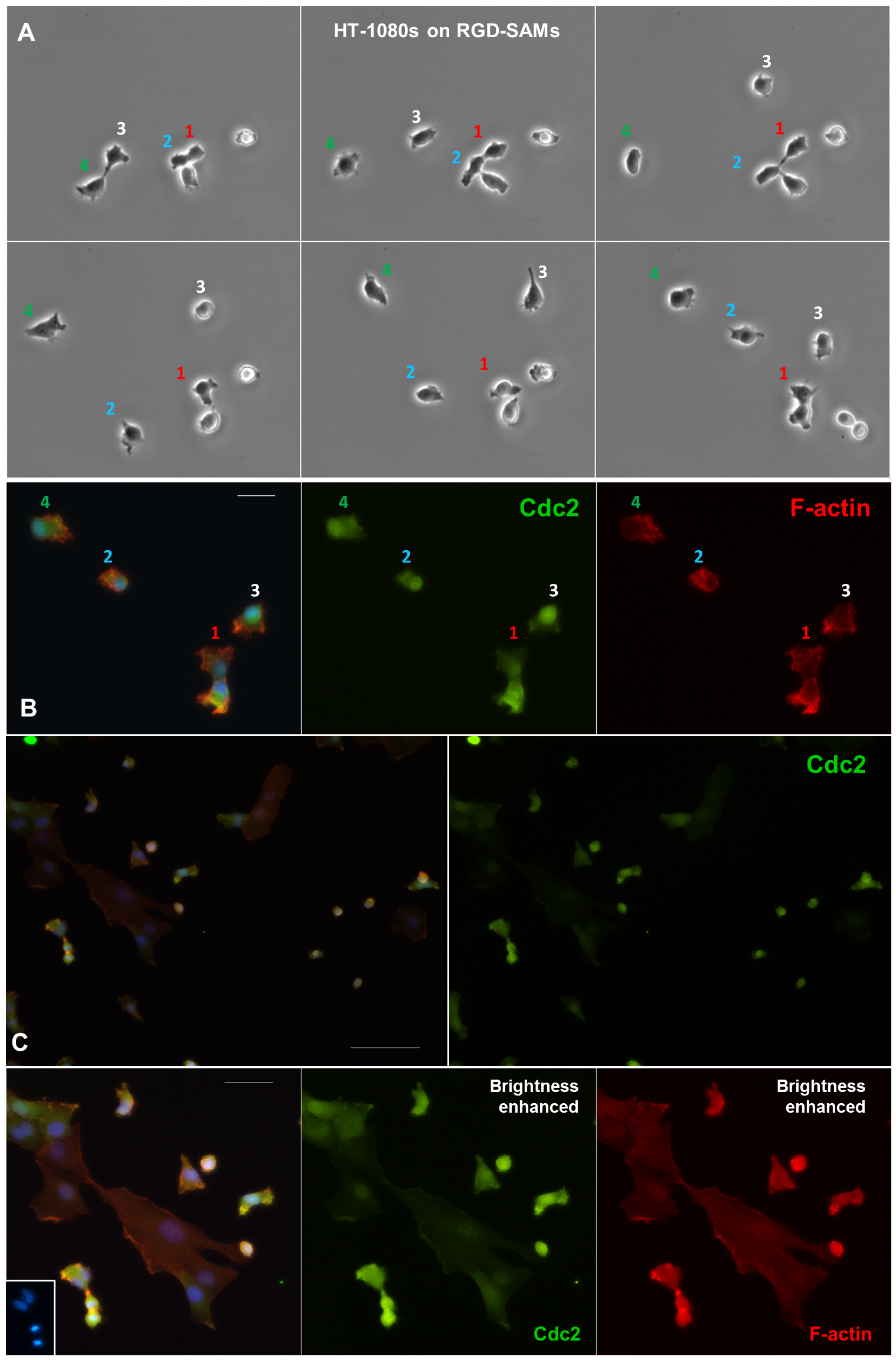

Supplement: Figure S10 — Cdc2 expression for HT-1080s on RGD-SAMs. (A) Time-lapse images (15 min / frame) illustrating HT-1080s on an RGD-SAM surface (1.7% mol fraction RGD). (B,C) Immunofluorescence images illustrating Cdc2 (Green), counterstained with TRITC-conjugated phalloidin (F-actin, red) and DAPI (nucleus, blue). Cells in (A) were fixed and are shown in (B). Images in (C) provide a broad overview for rounded and spread HT-1080s. Inset in (C) illustrates nuclei (DAPI) for cells during mitosis. (TIF) [file pone.0081689.s010.tif]

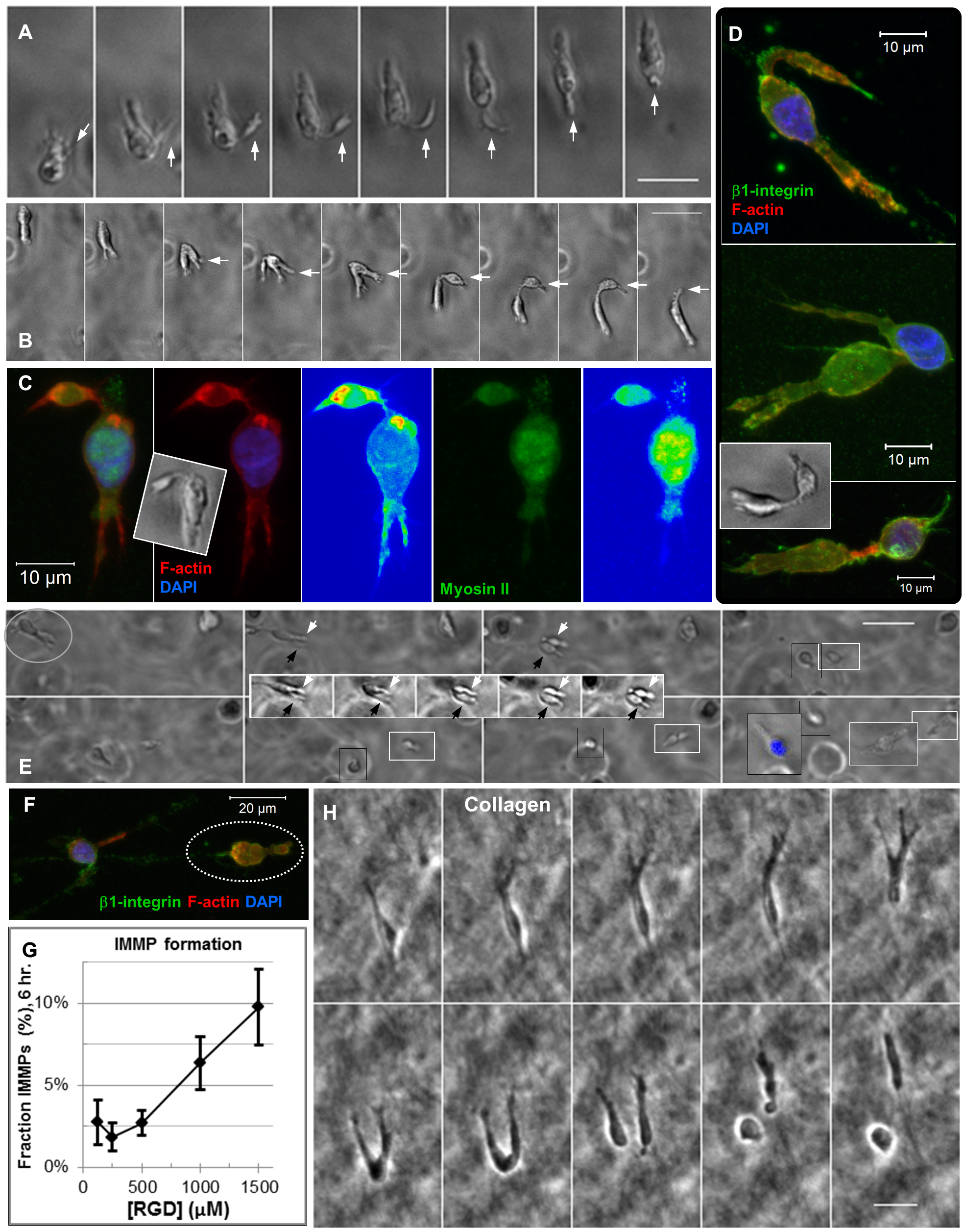

Supplement: Figure S11 — HT-1080s adopt unstable phenotypes in 3D matrices. (A,B) HT-1080s formed pronounced secondary protrusions (white arrows) that eventually retracted into the rear of the cell (See Movie S19). (C) Z-projected immunofluorescence images (Zeiss LSM Image Browser) for an HT-1080 in synthetic ECM (220 Pa, 1000 μM CRGDS) illustrating: Myosin IIb (green), counterstained with TRITC-conjugated phalloidin (F-actin, red) and DAPI (nucleus, blue). Rainbow intensity images are shown (to the right of false color images) to aid visualization of myosin IIb in the retracting feature at the rear of the cell. (D) Three separate z-projected immunofluorescence images (Zeiss LSM Image Browser) for HT-1080s in synthetic ECM (220 Pa, 1000 μM CRGDS) illustrating secondary features of various sizes; β1-integrin (green), counterstained with TRITC-conjugated phalloidin (F-actin, red) and DAPI (nucleus, blue). (E) Time-lapse images (1 hour / frame, inset = 15 min / frame; also Movie S17) illustrating formation of an “independent motile microplast” (IMMP, see Yount et. al, J. Neuro.-Oncol. 2007). Hydrogel was fixed and stained with DAPI (insets, final frame) to demonstrate that a nucleus was absent in the IMMP. (F) Immunofluorescence images illustrating an HT-1080 and an apparent IMMP in synthetic ECM (220 Pa, 1000 μM CRGDS); β1-integrin (green), counterstained with TRITC-conjugated phalloidin (F-actin, red) and DAPI (nucleus, blue). Image suggests that the IMMP migrated prior to fixing due to a trail of β1-integrin leading away from the HT-1080 containing a nucleus. Anucleate features of various sizes were common for HT-1080s, although motility cannot be definitively determined for IF images alone. (G) The fraction of HT-1080s characterized by abnormal fragmenting consistent with IMMP formation was proportional to RGD concentration (above 125 μM CRGDS). Cells were analyzed during the same 6 hour time period used for quantifying migration and cell division. Apparent IMMPs were determined based on c [file pone.0081689.s011.tif]
